# Polygenic Associations Between Motor Behavior, Neuromotor Traits, and Active Music Engagement in Four Cohorts

**DOI:** 10.1111/nyas.70191

**Published:** 2026-02-18

**Authors:** Tara L. Henechowicz, Peyton L. Coleman, Daniel E. Gustavson, Yasmina N. Mekki, Srishti Nayak, Rachana Nitin, Alyssa C. Scartozzi, Earvin S. Tio, Rivka T. N. van Klei, Daniel Felsky, Michael H. Thaut, Reyna L. Gordon

**Affiliations:** ^1^ Music and Health Science Research Collaboratory, Faculty of Music University of Toronto Toronto Ontario Canada; ^2^ Krembil Centre for Neuroinformatics Centre for Addiction and Mental Health Toronto Ontario Canada; ^3^ Vanderbilt Genetics Institute Vanderbilt University Medical Center Nashville Tennessee USA; ^4^ Music Cognition Laboratory, Department of Otolaryngology–Head and Neck Surgery Vanderbilt University Medical Center Nashville Tennessee USA; ^5^ Institute for Behavioral Genetics University of Colorado Boulder Boulder Colorado USA; ^6^ Center for Digital Genomic Medicine Vanderbilt University Medical Center Nashville Tennessee USA; ^7^ Department of Psychiatry University of Toronto Toronto Ontario Canada; ^8^ Division of Biostatistics, Dalla Lana School of Public Health University of Toronto Toronto Ontario Canada; ^9^ Rotman Research Institute Baycrest Hospital Toronto Ontario Canada; ^10^ Department of Anthropology University of Toronto Toronto Ontario Canada; ^11^ Temerty Faculty of Medicine University of Toronto Toronto Ontario Canada; ^12^ Vanderbilt Brain Institute Vanderbilt University Nashville Tennessee USA; ^13^ Department of Psychology Vanderbilt University Nashville Tennessee USA

**Keywords:** BioVU, brain structure, CLSA, individual differences, motor function, musicality, music training, polygenic scores, WLS

## Abstract

Active music engagement, that is, playing a musical instrument or singing, may be protective of motor function decline in aging. Although playing a musical instrument may transfer to benefits in motor function, it is also possible that the genetic architecture of motor behavior and the motor system brain structures may influence active music engagement. This study investigated whether polygenic scores (PGSs) for five behavioral motor traits, 12 structural brain traits, and seven rate‐of‐change in brain structure traits trained from existing genome‐wide association studies predict active music engagement in four independent cohorts: the Canadian Longitudinal Study on Aging (CLSA; *N* = 22,198), Wisconsin Longitudinal Study (WLS; *N* = 4605), Vanderbilt's BioVU Repository (BioVU; *N* = 6150), and Vanderbilt's Online Musicality Study (OM; *N* = 1559). Results were meta‐analyzed for each PGS main effect across outcomes and cohorts, revealing that PGS for a faster walking pace was associated with higher amounts of active music engagement. Within CLSA, a higher PGS for walking pace was associated with greater odds of engaging with music. Our findings suggest a shared genetic architecture between motor function and active music engagement. Future research should consider the genetic underpinnings of motor behavior when evaluating the effects of music engagement on motor function.

## 1 Introduction

Actively engaging with music, that is, playing a musical instrument or singing, is an underappreciated lifestyle factor in healthy aging because it is cognitively stimulating, involves motor learning, coordination, and multisensory integration [1]. Research suggests that skills gained from playing instruments or singing may transfer to enhanced skills in a nonrelated domain (e.g., cognitive or motor skills) and changes in brain structure and function [1, 2, 3]. Motor function is an integral part of neurocognitive health in aging, where motor decline precedes cognitive decline [4, 5]. There are several lines of phenotypic evidence showing how active music engagement may be protective of motor function. For example, a neuroimaging meta‐analysis showed that musicians, compared to nonmusicians, have greater auditory‐motor network connectivity and structural adaptations in regions important for motor control, including the corpus callosum, internal capsule, and sensorimotor and subcortical areas [6]. Behaviorally, musicians, compared to nonmusicians, have enhanced musical motor skills (e.g., audio‐motor synchronization) [7, 8, 9], better performance on standardized motor function assessments [10, 11], faster reaction time in spatial [12], multisensory integration [13], and visuomotor tasks [14], and enhanced motor sequence learning and retention [15, 16]. Music training interventions may also heighten motor skill development in children and adolescents [17, 18]. Given what is now known about genetic contributions to brain development and plasticity [19, 20, 21], it is possible that these transfer effects are partially influenced by shared genetic variation [22]. The current investigation seeks to understand how genetic factors for motor behavior and neuromotor traits may influence individual differences in music engagement.

Given the clear behavioral and neural links between musical instrument training and the motor system, several authors hypothesize that long‐term or regular playing of an instrument or singing is likely an environmental factor that modulates motor system neural plasticity [23]. In parallel, prior individual differences in motor skills or neuromotor features may contribute to who seeks out musical training [2, 3, 24], and prior work does not rule out the possibility that alternative or additional shared biology, that is, shared genetic architecture, underlies these phenotypic correlations. For example, the potential transfer of childhood musical instrument engagement to verbal ability 4 years later in adolescence is in part driven by shared genetic effects [25]. To investigate the hypothesized shared genetic architecture between active music engagement and motor phenotypes, we can adapt insights from a recent musicality cross‐trait framework, the Musical Abilities, Pleiotropy, Language, and Environment (MAPLE) [22]. The MAPLE framework makes a case that both musical abilities and musical environments may arise from genetic effects, that is, the niche‐picking phenomenon, where individuals are drawn to environmental influences for which they have a higher genetic propensity to be well adapted. Second, commonly found phenotypic associations between musicality and motor traits can be explained through partially shared genetic architecture and mediating neurobiological phenotypes [22].

Indeed, active music engagement and motor traits are moderate to highly heritable. Music‐related traits have an average heritability of 42% [26], including 78% for musical instrument engagement [25], 33–66% for musical aptitude [27], and 41–69% for musical practice [28, 29]. Recent genomic studies show evidence for SNP‐based heritability of 12% for music engagement [30]. For motor traits, a twin study found evidence for 68% and 70% heritability of motor control and motor learning, respectively [31]. Additionally, genetic studies of neurobiological endophenotypes, for example, the thickness, volume, and change in volume/thickness of motor system brain structures, are available publicly through Enhancing Neuroimaging Genetics through Meta‐analysis Consortium's genome‐wide association studies (GWASs) and show evidence for the heritability and detectable polygenic signals [19, 32, 33]. Accordingly, cross‐trait population‐level genomic methods, that is, polygenic scores (PGSs), can leverage existing GWASs for behavioral motor and neuromotor phenotypes to understand their shared biological etiology with active music engagement.

In this preregistered study (https://doi.org/10.17605/OSF.IO/SQ2NC), we examined whether PGSs for three categories of motor traits—behavioral, brain structure, and rate‐of‐change of brain structure—predict active music engagement in four cohorts: the Canadian Longitudinal Study on Aging (CLSA) [34, 35], Vanderbilt's BioVU repository (BioVU) [36, 37], Wisconsin Longitudinal Study (WLS) [38], and Vanderbilt's Online Musicality Study (OM) [30]. We predicted that greater genetic predisposition for motor function (i.e., greater PGS for walking pace, lower PGS for reaction time, lower PGS for hand muscle weakness, lower PGS for clinical diagnosis of Parkinson's disease, and lower PGS for motor coordination difficulties in childhood) would each be associated with higher amounts of music engagement. For neuromotor phenotypes, we predicted associations between genetic predispositions for brain structure and its rate‐of‐change in brain structure with active music engagement, without specific directional hypotheses.

## 2 Methods

### 2.1 Data

#### 2.1.1 Study Populations (Target Cohorts)

Four large cohort studies were analyzed: CLSA (*N* = 22,198), WLS (*N* = 4605), BioVU (*N* = 6150), and OM (*N* = 1559). Given limited data availability in these cohorts, analyses were constrained to individuals of European ancestry (see Supplementary Methods 1.1−1.4 for phenotyping and quality control protocols). We extracted active music engagement phenotypes and categorized the outcomes into constructs including music practice, music achievement, and music engagement (see Table S1 and Supplementary Methods 1.1−1.4 for inclusion/exclusion criteria).

#### 2.1.2 Discovery GWAS Summary Statistics Used for PGS Construction

PGSs were constructed using summary statistics from published GWASs of motor behavior traits (five traits), structural motor brain traits (12 traits), and the rate‐of‐change of brain structure across the lifespan [19] (seven traits) (see Table S2 for descriptions of the discovery GWASs). All discovery GWAS samples were nonoverlapping with target cohorts and were of European ancestry to match the target cohorts’ ancestries to avoid population stratification issues [39].

#### 2.1.3 Motor Behavior Discovery GWASs

We selected motor behavior traits from the GWAS atlas (https://atlas.ctglab.nl/) GWAS catalogue (https://www.ebi.ac.uk/gwas/), and from a general literature search. Available GWASs included reaction time (in milliseconds) [40], self‐reported walking pace [41], hand muscle weakness (grip strength <30 kg in males and <20 kg in females) [42], and motor coordination difficulties in children (measured by a sum of four items of the Movement Assessment Battery for Children) [43]. Additionally, we included a GWAS of Parkinson's disease case status [44], given both the clinical manifestation of progressive motor decline and the biological mechanisms of dysregulation of the dopaminergic system in Parkinson's disease [45]. See Table S2 for further description of the discovery GWASs.

#### 2.1.4 Neuromotor Brain Structure Discovery GWASs

We selected recent neuroimaging GWASs from the ENIGMA consortium for PGS of structural brain traits [32, 33]. For subcortical regions, we selected four of seven structures [32], the nucleus accumbens, pallidum, putamen, and caudate, because of their importance for motor learning and music training [6, 46]. For cortical structures, we extracted seven of Grasby et al.’s meta‐GWASs that controlled for global cortical thickness [33]. Specifically, we selected overall cortical thickness and six cortical thickness phenotypes from regions essential to motor and sensory systems: the precentral, postcentral, inferior parietal, insula, middle temporal, and superior temporal gyri. We also included Tissink et al.’s GWAS of combined cerebellar gray matter and cerebellar white matter volume [47].

#### 2.1.5 Neuromotor Rate‐of‐Change in Brain Structure Discovery GWASs

Lastly, we calculated seven PGSs corresponding to average rate‐of‐change for seven structural brain phenotypes across the lifespan, based on GWAS summary statistics from Brouwer et al. [19]. These seven phenotypes were selected as those overlapping with cross‐sectional PGS calculated above and that had SNP‐based heritability >0: rate‐of‐change in total brain volume, total cerebellar white matter volume, total cortical gray matter volume, mean cortical thickness, pallidum volume, putamen volume, and nucleus accumbens volume. These summary statistics meta‐analyzed the effects of SNPs on the average rate‐of‐change in brain structure across multiple samples of different ages, that is, including developmental, adult, and aging cohorts. For our study, we extracted the summary statistics that assumed the SNP effect sizes were consistent across the lifespan (i.e., independent of age), using standard meta‐GWAS approaches. Therefore, positive PGSs represent a genetic liability for positive change (more growth or less shrinkage) across the lifespan, and negative PGSs represent negative change (less growth or more shrinkage) across the lifespan.

#### 2.1.6 PGS Construction

PGSs were calculated using PRS‐CS or PRS‐CS‐auto, which use a Bayesian regression framework and place a continuous shrinkage prior on SNP effect sizes [48], outperforming traditional clumping and thresholding methods [49]. All GWAS summary statistics accessed were in GRCh37/hg19. Prior to calculation, PRS‐CS automatically handles allele and strand flips when aligning GWAS summary statistics, the 1.2 million SNPs in the reference panel, and the target datasets. We used PRS‐CS‐auto to construct PGSs for the GWAS summary statistics with *N* > 200,000 and PRS‐CS with phi = 0.01 for smaller sample sizes (i.e., GWASs for neuroimaging traits and motor coordination difficulties in children) as suggested for highly polygenic traits [48]. All PGSs were calculated with default parameters, *a* = 1 and *b* = 0.5 [48], and with the 1000 Genomes Project Phase 3 European linkage disequilibrium reference panel [48, 50].

### 2.2 Statistical Analysis

#### 2.2.1 Descriptive Statistics

All statistical analyses were performed using R statistical software v4.0.2. We reported descriptive statistics of age, sex, and available socioeconomic indicators for all four target cohorts in Table 1. For CLSA, WLS, and OM, we evaluated associations between active music engagement and education levels. For BiovU, we extracted the Area Deprivation Index, a ranking of neighborhoods by socioeconomic conditions in the United States that accounts for multiple domains of factors, including income, education, employment, and housing quality, to inform health delivery and policy [51, 52]. For cohorts with case‐control outcomes, we examined for associations between active music engagement outcomes and socioeconomic indicators using chi‐squared tests for categorical education variables and two‐sided *t‐*tests for continuous education variables. For OM, we investigated Spearman's rank correlations between all three active music engagement outcomes and education levels. Additionally, we reported correlations between age, sex, and all 24 PGSs within each cohort (see Figure S1A–D).

#### 2.2.2 Statistical Models

##### 2.2.2.1 Single PGS Models

General linear models were used to test for the main effects of each motor PGS on all music engagement outcomes within each cohort. For each outcome, 24 models were fit, each including one of the 24 motor PGSs with covariates of 10 genetic principal components, age, and biological sex, yielding 168 models. Continuous and binary outcomes were modeled with ordinary least squares linear and logistic regression, respectively. As preregistered, multiple testing corrections were applied to PGS main effect *p*‐values for the total number of models within each cohort (e.g., OM has 3 outcomes × 24 models for 72 tests; WLS has 2 outcomes × 24 models for 48 tests; BioVU has 1 outcome × 24 models for 24 tests; CLSA has 1 outcome × 24 models for 24 tests). Multiple test correction was assessed using the Benjamini−Hochberg false discovery rate (FDR) procedure, with a corrected *p*‐value (*q*FDR) of *q*FDR< 0.05 indicating a significant effect. We assessed model fit indices of *R* ^2^ for continuous outcomes and Nagelkerke‐Pseudo‐*R* ^2^ and *AUC* for binary outcomes. To evaluate out‐of‐sample performance and overfitting, we performed resampling analyses using 100 iterations of the 0.632 bootstrapping method commonly applied in GWASs [53, 54].

In secondary analyses, we fitted the same 168 models with PGS‐by‐sex interactions, applying the same multiple‐test correction approach. Additionally, we conducted sensitivity analyses using an influential observations approach wherein we identified influential observations using Cook's distance (threshold of > 4/*N*‐*k*‐1) and reproduced all 168 single PGS models with influential observations removed.

##### 2.2.2.2 Meta‐Analyses of PGS Models

We conducted meta‐analyses of the effect sizes for each of the 24 PGS associations across cohorts. Since outcomes were continuous and dichotomous, we converted log odds ratios to standard mean differences prior to meta‐analysis [55]. Inverse‐variance weighted random mixed‐effect meta‐analyses were conducted using the restricted maximum likelihood estimator with 150 maximum iterations from the *metafor* package in R [56]. For each of the 24 meta‐analyses, we evaluated Cochrane's *Q‐*statistic to determine if there was significant evidence for heterogeneity between studies. For sensitivity analyses, we conducted “leave‐one‐cohort‐out” analyses to assess whether the effect sizes and *p‐*values changed when any one of the cohorts was removed. In other words, we conducted a total of five sets of 24 meta‐analyses, initially including all four cohorts and subsequently removing each one of the four cohorts. Multiple testing correction using the FDR procedure was applied separately to each of the five sets of 24 meta‐analyses.

## 3 Results

### 3.1 Descriptive Statistics

**FIGURE 1:**
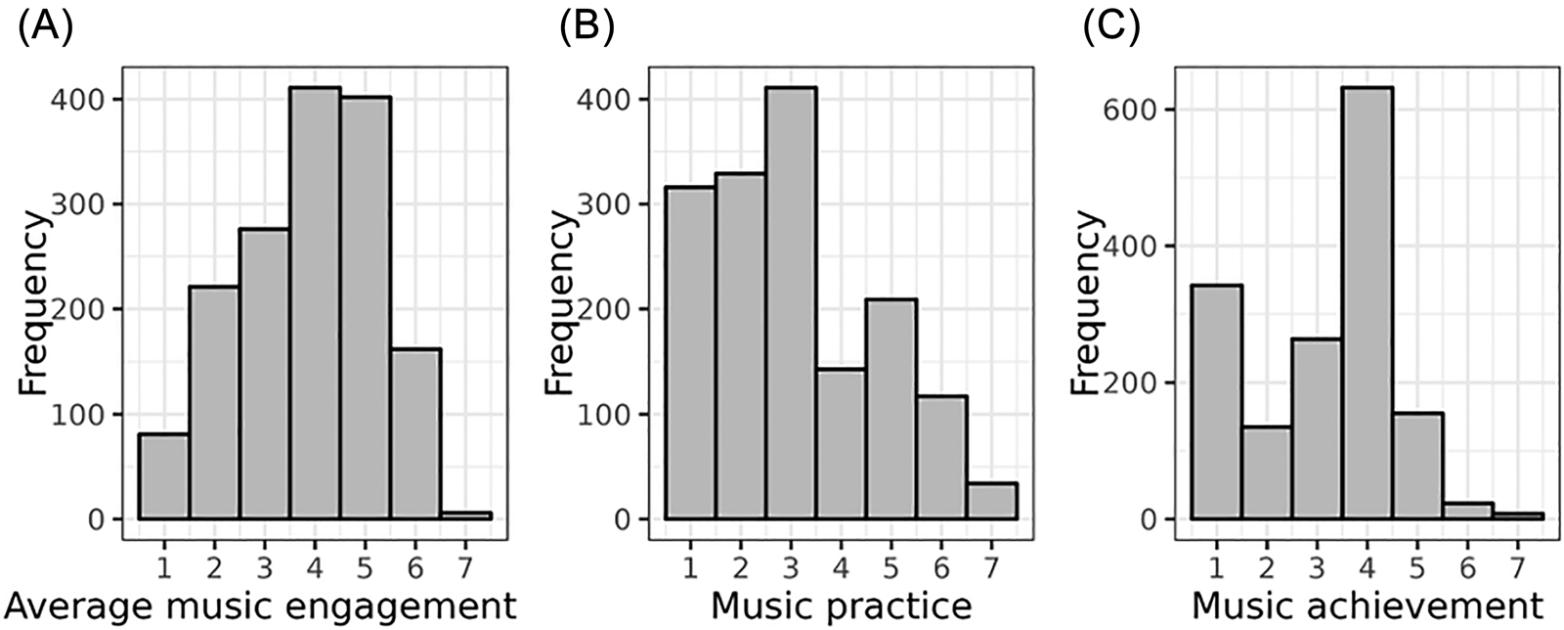
Distributions of continuous outcomes from Vanderbilt's Online Musicality Study (OM). (A) The distribution of average music engagement score, (B) music practice (as defined by question 33 in the gold‐MSI), and music achievement (as defined by the creative achievement questionnaire). See Supplementary Methods 1.4 for further phenotyping definitions in the OM cohort. Participants’ mean average score for music engagement was 3.99 ± 1.36, and the median was 4.25 (IQR = 3.0, 5.0). The median self‐report music practice hours was 3, corresponding to 1 h per day (IQR = 2, 4). The median of self‐report music achievement was 4, corresponding to “I have played or sung, or my music has been played in public concerts in my hometown, but I have not been paid for this” (IQR = 2, 4). All three music engagement phenotypes were significantly positively correlated: *r_s_* = 0.77 (*p*< 0.001) for average music engagement and music practice, *r*_s_ = 0.84 (*p*< 0.001) for average music engagement and music achievement, and *r*_s_ = 0.60 (*p*< 0.001) for music achievement and music practice.

Table 1 has descriptive statistics for *N* = 22,198 unrelated individuals from CLSA (62.99 ± 10.15 years old, 50% female, 77.55% had completed a post‐secondary degree/diploma), *N* = 4605 from WLS (64.22 ± 2.50 years old, 51% female, and the average education was 13.89 ± 2.40 years), *N* = 6150 from BioVU (53.13 ± 16.38 years old, 41% female, mean Area Deprivation Index of 0.33 ± 0.12), and *N* = 1559 from OM (45.85 ± 16.33 years old, 74% female, 79.74% had at least a bachelor's degree or equivalent). Distributions of the continuous outcomes in OM are shown in Figure 1.

**TABLE 1:** Descriptive statistics for target cohorts.

*Canadian Longitudinal Study on Aging*					
Characteristic	*n* for nonmissing	Overall sample, *N*=22,198	Several times a year or more frequently, *N* = 4556	Once a year or less, *N*=17,642	Group difference
		*n (%)*	*n (%)*	*n (%)*	*p‐*value
*Sex* [self‐report]	22,198				***p* = 0.02**
Female		11,153 (50.24%)	2219 (48.71%)	8934 (50.64%)	
Male		11,045 (49.76%)	2337 (51.29%)	8708 (49.36%)	
*Education*	22,161				***p* < 0.001**
Less than secondary school graduation		1171 (5.28%)	152 (3.34%)	1019 (5.79%)	
Secondary school graduation, no post‐secondary		2127 (9.60%)	264 (5.80%)	1863 (10.58%)	
Some post‐secondary education		1677 (7.57%)	280 (6.15%)	1397 (7.93%)	
Post‐secondary degree/diploma		17,186 (77.55%)	3856 (84.71%)	13,330 (75.70%)	
		M (SD)	M (SD)	M (SD)	
*Age* [years]	22,198	62.99 (10.15)	62.21 (10.08)	**63.18 (10.16)**	***p* < 0.001**

*Wisconsin Longitudinal Study*					
Characteristic	*n* for nonmissing	Overall sample, *N*=4605	Played a musical instrument at 35 often or sometimes *N* = 1130	Played a musical instrument at 35 never *N* = 3475	Group difference
		*n (%)*	*n (%)*	*n (%)*	*p‐*value
*Sex* [self‐report]	4605				***p*< 0.001**
Female		2348 (50.99%)	720 (63.72%)	1628 (46.85%)	
Male		2257 (49.01%)	410 (36.28%)	1847 (53.15%)	
		M (SD)	M (SD)	M (SD)	
*Age* [years]	4605	64.22 (2.50)	64.15 (2.33)	64.24 (2.55)	*p* = 0.25
*Education* [years]^a^	4592	13.69 (2.40)	14.45 (2.53)	13.71 (2.34)	***p <* 0.001**

*Vanderbilt's BioVU Repository*					
Characteristic	*n* for nonmissing	Overall sample, *N* = 6150	Musically active patient cases, *N* = 1259	Population‐matched controls, *N* = 4891	Group difference
		*n (%)*	*n (%)*	*n (%)*	*p‐*value
*Gender* [self‐report]	6150			***p =* 0.01**	***p =* 0.01**
Female		2515 (40.89%)	553 (43.92%)	1962 (40.11%)	
Male		3635 (59.11%)	706 (56.08%)	2929 (59.89%)	
		M (SD)	M (SD)	M (SD)	
*Age* [median age at record in years]	6150	53.13 (16.38)	53.07 (16.52)	53.15 (16.34)	***p* = 0.87**
*Area Deprivation Index*	5265	0.33 (0.12)	0.31 (0.12)	0.33 (0.12)	***p* < 0.001**

*Vanderbilt's Online Musicality Study*					
Characteristic	*n* for nonmissing	European subset *N* = 1559	Association with music engagement outcomes (*r_s,_ p‐*value)		
		*n (%)*	*Average music engagement*	*Practice*	*Achievement*
*Gender* [self‐report]	1559				
Female		**1146 (73.51%)**	***t =* −2.6,** **(*p =* 0.01)**	***t =* −3.0,** **(*p =* 0.003)**	*t =* −0.6, (*p =* 0.56)
Male		413 (26.49%)			
*Education*	1555		***r*_s_ = 0.08 (*p* = 0.003)**	*r*_s_ = 0.03 (*p =* 0.19)	***r*_s_ *= 0*.11 (*p* < 0.001)**
Less than high school		2 (0.13%)			
High school (or equivalent)		46 (2.96%)			
Some college or associate's degree		267 (17.17%)			
Bachelor's or equivalent		553 (35.56%)			
Master's or equivalent		510 (32.80%)			
Doctorate or equivalent		177 (11.38%)			
		M (SD)			
*Age* [years]	1559	45.85 (16.33)	***r =* −0.20** **(*p* < 0.01)**	***r =* −0.20** **(*p* < 0.01)**	***r =* −0.13** **(*p* < 0.01)**

*Note*: Bold denotes significant group differences in socioeconomic and education variables between cases and controls.

### 3.2 Single PGS Model Results

#### 3.2.1 Main Effects of Motor Behavior PGSs on Music Engagement Outcomes

PGS for walking pace was positively associated with greater odds of music engagement only in CLSA (OR = 1.07, 95% CI [1.03, 1.10], *p*< 0.001, *q*FDR = 0.002) (see Figure 2A). At an uncorrected threshold of *p*< 0.05, PGS for walking pace was associated with greater odds of music engagement in BioVU (OR = 1.08, 95% CI [1.02, 1.15], *p* = 0.01, *q*FDR = 0.13). At an uncorrected threshold of *p*< 0.05, there was suggestive evidence that higher PGS for reaction time (i.e., slower reaction time) was associated with lower odds of music engagement in CLSA (OR = 0.97, 95% CI [0.94, 1.00], *p* = 0.04, *q*FDR = 0.33) and in BioVU (OR = 0.92, 95% CI [0.86, 0.98], *p* = 0.007, *q*FDR = 0.13). See Table S3 for all main effects and bootstrapped performance indices and Table S4 for sensitivity analysis results.

FIGURE 2Forest plot of 24 PGS main effects and meta‐analyses across outcomes. All effects arepresented in a forest plot as effect sizes and 95% confidence intervals. Meta‐analyzed effects are represented as diamonds. For continuous outcomes, main effects were estimated using linear regression and are represented as an effect size. For case‐control or dichotomized outcomes, logistic regression models were used to calculate log odds, which were then transformed into standard mean differences. The results are paneled by PGS category (A) motor behavior (five PGSs), (B) neuromotor brain structure (12 PGSs), and (C) neuromotor rate‐of‐change in regional volume/thicknesses (seven PGSs). Asterisks (*) denote significant meta‐effects after multiple‐test correction (*q*FDR*<* 0.05). See Table S6 for the source data for plotting. Abbreviations: BioVU, Vanderbilt's BioVU Repository; CLSA, Canadian Longitudinal Study on Aging; OM, Vanderbilt Online Musicality Study; PGS, polygenic score; ROC, rate‐of‐change; Thick., thickness; Vol., volume; WLS, Wisconsin Longitudinal Study.

**Figure:**
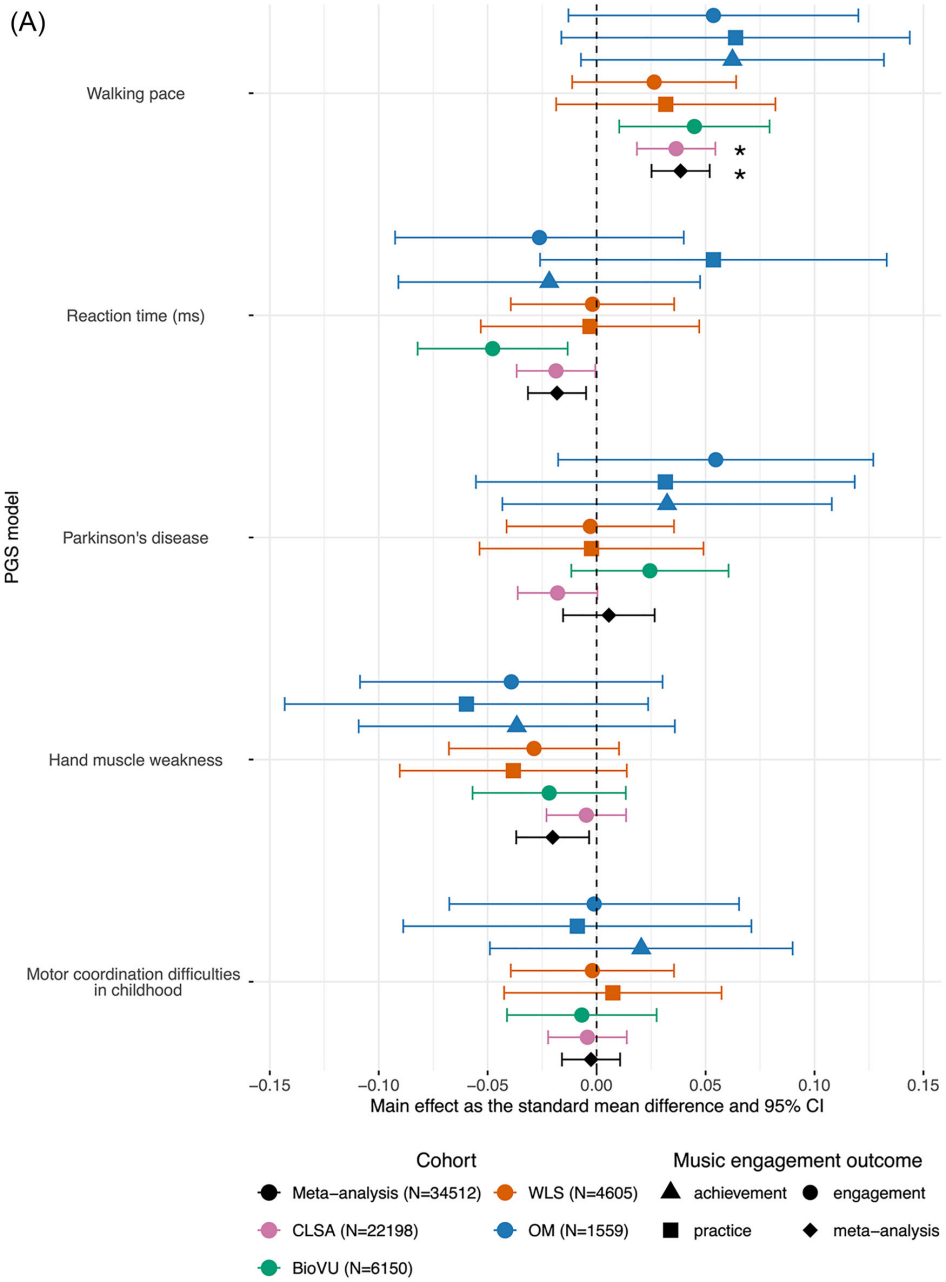

**Figure:**
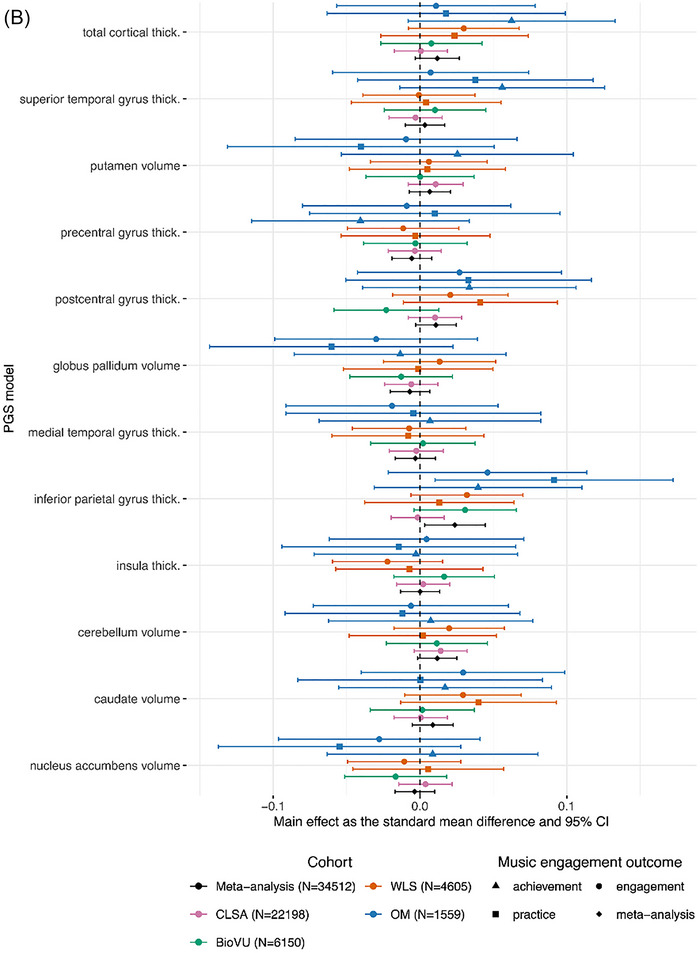

**Figure:**
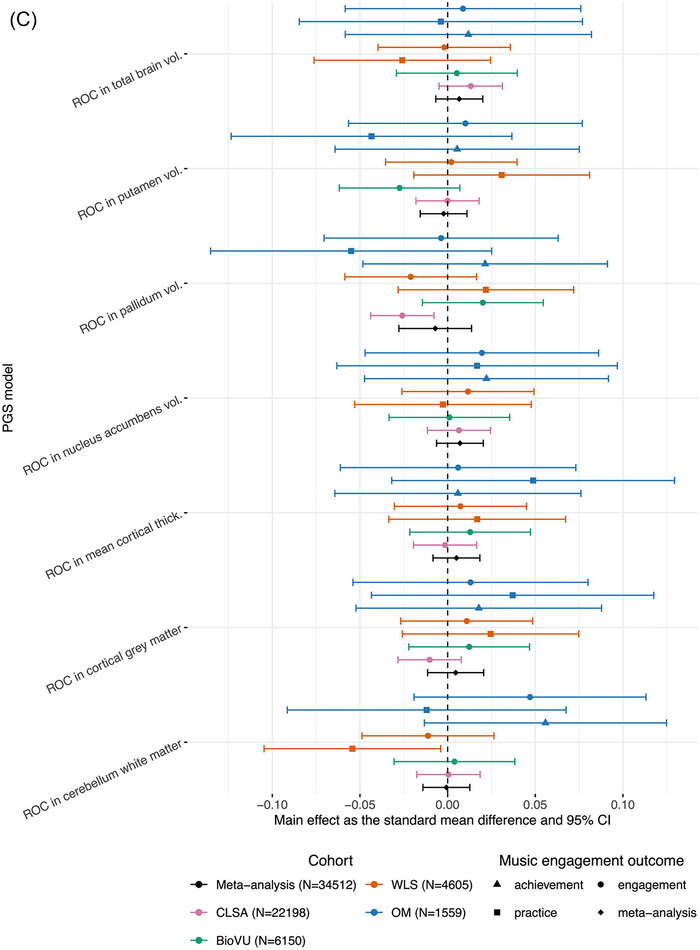

#### 3.2.2 Main Effects of Structural Brain PGSs on Music Engagement Outcomes

At an uncorrected threshold of *p*< 0.05, PGS for inferior parietal gyrus thickness was positively associated with music practice in OM (*b =* 0.09, 95% CI [0.01, 0.17], *p* = 0.03, *q*FDR = 0.93) (see Figure 2B).

#### 3.2.3 Main Effects of Neuromotor Rate‐of‐Change PGSs on Music Engagement Outcomes

At an uncorrected threshold of *p*< 0.05, there were nominal associations between neuromotor rate‐of‐change PGSs and music engagement, although there was no consistency across cohorts (see Figure 2C). In CLSA, PGS for the rate‐of‐change in pallidum volume was negatively associated with odds of being a musically active case (OR = 0.95, 95% CI [0.92, 0.99], *p =* 0.005, *q*FDR = 0.06), suggesting that engaging with music is associated with a genetic predisposition for more shrinkage of the pallidum. In WLS, PGS for the rate‐of‐change in cerebellum white matter volume was associated with decreased odds of practicing a musical instrument (OR = 0.91, 95% CI [0.83, 0.99], *p =* 0.03, *q*FDR = 0.90), that is, a greater likelihood of music practice was associated with a higher genetic predisposition for more shrinkage of the cerebellum white matter.

#### 3.2.4 Interaction Effects of Sex on PGS Models

There were no significant sex‐by‐PGS interactions after multiple testing corrections (see Table S5). However, at the suggestive *p<* 0.05 threshold, there were some potential interaction effects of sex and PGS for walking pace in two cohorts. In CLSA, there was an interaction between PGS for walking pace and sex predicting music engagement in CLSA (OR = 0.92, 95% CI [0.86, 0.98], *p* = 0.014, *q*FDR = 0.34). Greater PGS for walking pace was associated with greater odds of music engagement in females (OR = 1.11, 95% CI [1.06, 1.17], *p*< 0.001), although not significant in males (*p*> 0.05) (see Figure S2). For all three outcomes in OM, the PGS for walking pace had a significant interaction with sex at the suggestive threshold, showing positive associations between PGS for walking pace and music engagement in females but not males. There was an interaction between PGS for walking pace and sex predicting music engagement in OM (*b* = −0.21, 95% CI [−0.36, −0.05], *p* = 0.008, *q*FDR = 0.29). The PGS for walking pace was associated with increased music engagement in females (*b* = 0.1, 95% CI [0.03, 0.18], *p* = 0.007), although not significant in males (*p*> 0.05) (see Figure S3). When predicting music practice, there was an interaction between PGS for walking pace and sex (*b* = −0.26, 95% CI [−0.45, −0.08], *p* = 0.005, *q*FDR = 0.29). The PGS for walking pace was associated with increased music practice in females (*b* = 0.13, 95% CI [0.04, 0.22], *p* = 0.006), although not significant in males (*p*> 0.05) (see Figure S4). When predicting music achievement, there was an interaction between PGS for walking pace and sex (*b* = −0.19, 95% CI [−0.36, −0.03], *p* = 0.018, *q*FDR = 0.45). The PGS for walking pace was associated with increased music achievement in females (*b* = 0.11, 95% CI [0.03, 0.19], *p* = 0.007), although not significant in males (*p*> 0.05) (see Figure S5). To summarize, associations between PGS for walking pace and music engagement were stronger in females compared to males in OM and CLSA.

Additionally, some trends showed interactions between neuromotor trait PGSs and sex in all four cohorts. In BioVU, there was a significant interaction between the PGS for the rate of change in total brain volume and sex (OR = 1.16, 95% CI [1.03, 1.32], *p* = 0.02, *q*FDR = 0.38), although marginal effect models were not significant in males or females (see Figure S6). In CLSA, the model for PGS for insula thickness had a significant interaction with sex (OR = 1.07, 95% CI [1.00, 1.14], *p* = 0.04, *q*FDR = 0.44). However, marginal effect models were not significant in males or females (see Figure S7). In WLS, the interaction model for the PGS for the rate‐of‐change in cerebellum white matter predicting music practice had a significant interaction with sex (OR = 1.22, 95% CI [1.01, 1.48], *p* = 0.04, *q*FDR = 0.89). The PGS for the rate‐of‐change in cerebellum white matter was associated with decreased odds of practicing a musical instrument in females (OR = 0.84, 95% CI [0.75, 0.95], *p* = 0.003) but not in males (*p*> 0.05) (see Figure S8). In OM, there was an interaction between the PGS for the rate‐of‐change of mean cortical thickness and sex predicting music practice (*b* = 0.20, 95% CI [0.02, 0.38], *p* = 0.03, *q*FDR = 0.50). However, marginal effect models were not significant in males or females (see Figure S9). Together, results show potential evidence for sex differences in the genetic associations between neuromotor traits and music engagement, although there was no consistency across cohorts.

### 3.3 Meta‐Analyses of PGS Models

The meta‐analyzed PGS for walking pace was positively associated with active music engagement (*b =* 0.04, 95% CI [0.03, 0.05], *p*< 0.001, *q*FDR< 0.001) with no significant heterogeneity among studies (*Q*(6) = 1.68, *p* = 0.95) (see Figure 2A). The leave‐one‐cohort‐out analyses showed that these results were consistent regardless of any cohort being left out of the analyses. Even when the largest cohort, CLSA, was left out of the analyses, the results remained significant (*b* = 0.04, 95% CI [0.02, 0.06], *p* = 5.2 × 10^−5^, *q*FDR = 0.001).

At an uncorrected threshold of *p*< 0.05, PGS for reaction time was negatively associated with active music engagement (*b* = −0.02, 95% CI [−0.03, −0.005], *p* = 0.008, *q*FDR = 0.09) with no significant heterogeneity among studies (*Q*(6) = 7.10, *p* = 0.31) and PGS for hand muscle weakness was negatively associated with active music engagement (*b* = −0.02, 95% CI [−0.04, −0.003], *p* = 0.02, *q*FDR = 0.14) with no significant heterogeneity among studies (*Q*(6) = 4.46, *p* = 0.61) (see Figure 2A). Additionally, PGS for inferior parietal gyrus thickness was positively associated with active music engagement (*b =* 0.02, 95% CI [0.003, 0.04], *p* = 0.02, *q*FDR = 0.14) with no significant heterogeneity among studies (*Q*(6) = 9.44, *p* = 0.15) (see Figure 2B). Additionally, the meta‐analyses for the inferior parietal gyrus PGS and the hand muscle weakness PGS were consistent when CLSA was left out of the meta‐analysis (*b* = 0.03, 95% CI [0.01, 0.05], *p* = 0.0008, *q*FDR = 0.01 and *b* = −0.03, 95% CI [−0.05, −0.01], *p* = 0.002, *q*FDR = 0.02, respectively). This may suggest that associations for these PGSs were consistent across the other cohorts. See Table S7 for the complete results of the leave‐one‐cohort‐out meta‐analyses.

## 4 Discussion

Our investigation revealed insights into the potential shared genetic relationship between music engagement and motor traits. First, the genetic predisposition for enhanced motor function, that is, higher PGS for walking pace, was significantly associated with greater music engagement in CLSA and once meta‐analyzed across four cohorts. We also observed potential associations between greater PGSs for inferior parietal gyrus thickness, lower PGSs for hand muscle weakness, and lower PGSs for reaction time and more active music engagement. Despite this, the meta‐analyses and cohort‐level analyses provide inconclusive evidence as to whether PGSs for neuromotor traits predict active music engagement.

The meta‐analyses revealed that PGSs for walking pace were positively associated with active music engagement, with the strongest effect in CLSA. The genetics of walking pace are important for motor function and are connected to several domains of health. For example, faster self‐reported walking is causally associated with lower cardiac and stroke risk [41, 57] and genetically correlated with lower cardiometabolic, respiratory, psychiatric, and all‐cause mortality risk and higher educational attainment [41, 58].

We observed patterns showing that genetic associations between walking pace and active music engagement may differ between males and females, which is expected, given the role of sex differences in brain development and aging [59, 60, 61]. The meta‐analyses across cohorts also revealed patterns of association between PGS for hand muscle weakness and less active music engagement. Hand muscle weakness, measured by low hand grip strength, is a simple motor performance metric that provides a window into neural resources needed to commit a motor action, for example, hand grip strength recruits corticospinal tract motor neurons [62]. Hand grip strength also has genetic associations with frailty, cardiac, psychiatric, motor, and general health traits [63, 64, 65, 66, 67]. Niarchou et al. observed genetic correlations between grip strength, walking pace, and the GWAS musical rhythm, with a possible explanation being the relevance of walking pace to biological rhythms. Given that the GWASs of reaction time and Parkinson's disease discovery had large sample sizes, their limited prediction of active music engagement in this study may show that phenotypes are not as relevant as grip strength and walking pace.

Second, we also observed trends of associations between PGS for greater inferior parietal gyrus thickness and more active music engagement in the meta‐analysis, with the strongest association being with music practice in the OM cohort. The inferior parietal lobe is critical for music processing because of its role in sensorimotor integration [68] and musical rhythm processing [69, 70]. Despite the potential relationship between PGS for greater inferior parietal gyrus thickness and more music engagement, we did not observe significant meta‐analyzed or cohort‐level associations with other neuromotor traits.

Our results provide evidence for potential shared genetics between active music engagement and behavioral motor traits, suggesting that investigations of transfer from musical to nonmusical skills should consider individual differences. Several authors have suggested that genetic factors and gene‐by‐environment interactions likely confound the literature on the transfer to nonmusical domains [3, 24, 71, 72]. Schellenberg and colleagues have evaluated the state of the literature on music‐based transfer to nonmusical domains of cognition, suggesting that music‐induced benefits are weak correlations [3, 72, 73]. Individual differences (not limited to genetic predispositions) possibly magnify the observed correlations between music engagement and other domains [72]. Thus, gene‐by‐environment interactions can be incorporated into future music‐based intervention studies to improve their rigor.

Although our results showed potential evidence for associations between PGSs for reaction time and active music engagement, they were not significant after multiple testing correction. Prior work has shown that musicians, compared to nonmusicians, have enhanced reaction time in multisensory integration tasks [13, 68]. However, genetic evidence for shared genetic architecture of musical rhythm and reaction time is conflicting, that is, Niarchou et al. showed modest genetic correlations between musical rhythm [37] and Gustavson et al. showed no associations using PGS methods [30]. For Parkinson's disease, there is little evidence showing that being a musician is protective of Parkinson's disease, despite the therapeutic benefits of musical rhythm interventions for gait and motor function in Parkinson's disease patients. Likewise, PGSs for subcortical volumes, which are thought to be potential neuroendophenotypes correlated with genetic risk for Parkinson's disease [32], were also not predictive of active music engagement in the present study. Given that there is a lot of variation in motor decline in preceding a Parkinson's disease diagnosis [71], it is possible that the genetic factors influencing motor decline trajectories (rather than diagnoses) may be more relevant to active music engagement.

Our results suggest that PGSs for the rate‐of‐change in brain structure in motor regions do not account for differences in active musical engagement. However, Brouwer et al.’s age‐independent meta‐GWASs on the rate‐of‐change of brain structures capture the average rate‐of‐change across the lifespan rather than during specific developmental or aging periods. Future work should investigate PGSs that are age‐dependent and incorporate how SNP effects vary in size and direction across the lifespan. Moreover, our findings do not rule out the possibility that other plasticity traits may influence active music engagement. For example, the neural structure of white matter tracts, especially the right corticospinal tract organization, in infancy predicts rhythmic and musical abilities in school‐age children [74]. Future investigations should also consider functional neuromotor phenotypes, for example, the genetic architecture of functional brain networks [75], especially given the relevance of sensorimotor or audiomotor networks to active music engagement [6].

Although the meta‐analyses revealed promising insights, we discuss some study limitations. While BioVU and OM had validated outcomes, the outcomes in WLS and CLSA had unknown psychometric profiles. The phenotypes also included singing together with musical instrument engagement, which may require different neuromotor resources, for example, singing requires coordination of laryngeal, oropharyngeal, and facial muscles from the corticobulbar tract [76], and musical instruments require fine motor control of the upper limbs that descend from the corticospinal tract [2]. Therefore, motor PGSs and active music engagement associations may be deflated due to this heterogeneity. Current initiatives, that is, the Musicality Genomics Consortium, are tackling this issue by validating succinct measurements to implement in biobanks.

Second, the predictive power of PGSs is constrained by discovery and target cohort sample sizes and phenotype measurements [39], for example, the motor coordination difficulties in childhood and neuromotor discovery GWASs had smaller sample sizes than most of the behavioral motor GWASs. Additionally, current motor behavior GWASs do not capture the full range of complex motor phenotypes, but recent studies show the feasibility of collecting motor learning data from thousands of individuals online [77] for future GWASs. Lastly, due to data availability, our primary analyses were constrained to genetic similarity with European ancestry due to differences in allele frequencies and the limited generalizability of PGSs across ancestries [78, 79]. Future work should leverage multiancestry methods and focus on musicality phenotyping in diverse populations.

## 5 Conclusion

PGSs for higher self‐reported walking pace may be associated with greater active music engagement. However, analyses did not yield sufficient evidence to support that PGSs for neuromotor phenotypes predict active music engagement. Our results suggest that shared genetic factors for motor function may predict active music engagement, holding significance for longitudinal studies and interventions aiming to understand the transfer of musical learning to nonmusical domains.

## Author Contributions

T.L.H.: Data curation, research design, data cleaning and quality control of genetic data, conducted all analyses, writing of first draft, and writing of all sections on the manuscript; P.L.C.: Data curation (CLSA, WLS), data collection (OM), genetic quality control (OM), feedback on supplementary materials; D.E.G.: Data collection (OM), feedback on study design for the preregistration phase, and feedback on the manuscript. Y.N.M.: Study design, data curation, and genetic quality control support (WLS, CLSA, OM); S.N.: Data collection (OM) and manuscript feedback; R.N.: Data collection (OM) and genetic quality control (WLS, CLSA, OM); A.C.S.: Data collection (OM), code review, and manuscript feedback; E.S.T.: Genetic quality control support for CLSA and methods development for longitudinal summary statistics scoring; R.T.N.v.K.: Methods development for longitudinal summary statistics scoring; D.F.: Supervision, data curation (CLSA), study design, statistical analyses feedback, methods development for longitudinal summary statistics scoring, manuscript feedback and revisions; M.H.T: Funding, supervision, study design, manuscript feedback; R.L.G.: Funding, supervision, data curation (all cohorts), study design, analyses feedback, manuscript feedback and revisions.

## Conflicts of Interest

The authors have no conflicts of interest to declare.

## Disclaimers

The opinions expressed in this manuscript are the author's own and do not reflect the views of the Canadian Longitudinal Study on Aging, BioVU, or the Wisconsin Longitudinal Study. The content is solely the authors’ responsibility and does not necessarily represent the official views of the National Institutes of Health.

## Data and Code Availability Statement

Individual data are available from the Canadian Longitudinal Study on Aging (www.clsa‐elcv.ca) for researchers who meet the criteria for access to deidentified CLSA data. Individual data from the Wisconsin Longitudinal Study (https://wls.wisc.edu/) are available for researchers who meet the criteria to access deidentified data. Individual data from Vanderbilt's BioVU repository cannot be shared publicly due to patient confidentiality; however, Vanderbilt University Medical Center researchers may request access (victr.vumc.org). Individual deidentified data for Vanderbilt's Online Musicality will also be deposited to dbGaP. Study identifiers for all GWAS summary statistics used for polygenic score calculations are in Table S2. Source summary level data for all figures and the output of all models are available in the Supplementary Materials. Code for all modeling and figures are available on Open Science Framework (https://osf.io/jvsk7/overview).

## Supporting information



Supplementary Tables: nyas70191‐sup‐0001‐TablesS1‐S2.docx

Supplementary Tables: nyas70191‐sup‐0002‐TablesS3‐S7.xlsx

Supplementary Materials: nyas70191‐sup‐0003‐SuppMat.docx
