# Supplementary material for: Polygenic Associations Between Motor Behavior, Neuromotor Traits, and Active Music Engagement in Four Cohorts
Source: Ann N Y Acad Sci. 2026 Feb 18;1556(1):e70191. doi: 10.1111/nyas.70191 (PMC12916079; doi:10.1111/nyas.70191)
Supplement: Supplementary file 1 — Supplementary Tables: nyas70191‐sup‐0001‐TablesS1‐S2.docx [file NYAS-1556-0-s003.docx]

**Supplementary Table 1.** *Description of music engagement phenotypes in cohorts with individual genotyped data.*

| *Cohort* | *Construct* | *Data type* | *Original phenotype measurement* | *Transformation* | *N* |
| --- | --- | --- | --- | --- | --- |
| *BioVU* | Active music engagement | case-control | Musically active patients extracted from EHR compared to matched control patients  [(Niarchou et al., 2021)](https://www.zotero.org/google-docs/?2Ft1bp). | N/A | 6,150 |
| *CLSA* | Active music engagement | case-control | “About how much time do you spend doing each of the following activities, taking into account both work and leisure time?” for “Playing a musical instrument or singing in a choir” Everyday, Several times a week, Several times a month, Several times a year, Once a year or less (GEN_MUSC_COM from Comprehensive Dataset version 7.0) | Dichotomized to several times a year or greater = 1 vs. once a year or less = 0. | 22,198 |
| *WLS* | Music engagement | case-control | q7spd_3: How often did you play a musical instrument when you were 35?  Often, sometimes, or never | Dichotomized to sometimes or often = 1 vs. never = 0 | 4,605 |
| *WLS* | Music practice | case-control | q7spd1: In the past year, how many hours per month did you play a musical instrument?  (continuous response in hours) | Dichotomized to >0 hours = 1 vs. 0 = 0. | 4,605 |
| *OM* | Music engagement | continuous | Summed score of 3 gold-MSI items and the 1 Creative Achievement Questionnaire item (Gustavson et al., 2023). | N/A | 1,559 |
| *OM* | Music achievement | continuous | “How engaged with music are you?” item with 7 levels from Creative Achievement Questionnaire [(Carson et al., 2005; Theorell et al., 2014)](https://www.zotero.org/google-docs/?4Aoyao). | N/A | 1,559 |
| *OM* | Music practice | continuous | Gold-MSI q33: At the peak of my interest, I practiced 0 / 0.5 / 1 / 1.5 / 2 / 3-4 / 5 or more hours per day on my primary instrument.  (Müllensiefen et al., 2014) | N/A | 1,559 |

*Note*. BioVU = Vanderbilt's biorepository of DNA extracted from discarded blood collected during routine clinical testing and linked to de-identified medical records in the Synthetic Derivative. In this case, we refer to the subset from Niarchou et al.’s (2021) application of a phenotyping algorithm to extract musically active patients from electronic health records. OM = Vanderbilt’s Online Musicality Study.

WLS = Wisconsin Longitudinal Study. CLSA = Canadian Longitudinal Study on Aging.

**Supplementary Table 2.** *List of discovery GWAS included in PGS analyses.*

| Construct | Measured phenotype | Study | pmid | Cohort | SNP-*h*^2^ | 95% CI | N | interpretation |
| --- | --- | --- | --- | --- | --- | --- | --- | --- |
| *motor behaviour* | | | | | | | | |
| Neuromuscular strength | Hand muscle weakness in older adults | Jones et al. (2021) | 33510174 | EUR, meta-analysis | 4.4% | (3.8%, 5.0%) | 256,523 | Lower PGS = better motor function |
| Processing speed | Reaction time (ms) | Neale Lab (2019) | N/A | UKBB, EUR | 8.1% | (7.5%, 87%) | 358,695 | Lower PGS = better motor function |
| Locomotion | Walking Pace (ordinal self-report question) adjusted for BMI | Timmins et al. (2020) | 33128006 | UKBB, EUR | 8.9% | (8.6% to 9.2%) | 450,967 | Higher PGS = better motor function |
| Motor coordination difficulties in childhood | Sum of | Mountford et al. (2021) | 34177493 | EUR | 26.1% | (5.8%, 46.3%) | 4,542 | Lower PGS = better motor function |
| Parkinson’s disease | cases and proxy cases vs. controls | Nalls et al. (2019) | 31701892 | EUR, meta-analysis | 22% | (18%, 26%) | 482,730 | Lower PGS = better motor function |
| *neuromotor phenotypes* | | | | | | | | |
| subcortical structure | Nucleus accumbens volume | Satizabal et al. (2019) | 31636452 | EUR, meta-analysis | 19.1% | (15.3%, 22.9%) | 28,697 | Higher PGS = greater volume |
| subcortical structure | Pallidum volume | Satizabal et al. (2019) | 31636452 | EUR, meta-analysis | 16.7% | (12.8%, 20.6%) | 30,142 | Higher PGS = greater volume |
| subcortical structure | Caudate volume | Satizabal et al. (2019) | 31636452 | EUR, meta-analysis | 25.6% | (21.7%, 29.5%) | 30,153 | Higher PGS = greater volume |
| subcortical structure | Putamen volume | Satizabal et al. (2019) | 31636452 | EUR, meta-analysis | 25.7% | (21.4%, 30.0%) | 29,984 | Higher PGS = greater volume |
| Cerebellar structure | Total cerebellar volume | Tissink et al. (2022) | 35842455 | EUR, UKBB | 39.8% | (33.6%, 46.0%) | 27,486 | Higher PGS = greater volume |
| cortical structure | Total mean cortical thickness | Grasby et al. (2020) | 32193296 | EUR, meta-analysis | 25.8% | (21.5%, 30.1%) | 30,879 | Higher PGS = greater thickness |
| cortical structure | Precentral gyrus thickness adjusted for global thickness | Grasby et al. (2020) | 32193296 | EUR, meta-analysis | 8.4% | (5.3%, 11.4%) | 32,185 | Higher PGS = greater thickness |
| cortical structure | Postcentral gyrus thickness adjusted for global thickness | Grasby et al. (2020) | 32193296 | EUR, meta-analysis | 9.6% | (6.5%, 12.7%) | 31,681 | Higher PGS = greater thickness |
| cortical structure | Inferior parietal gyrus thickness adjusted for global thickness | Grasby et al. (2020) | 32193296 | EUR, meta-analysis | 9.9% | (6.8%, 12.9%) | 32,850 | Higher PGS = greater thickness |
| cortical structure | Middle temporal gyrus thickness adjusted for global thickness | Grasby et al. (2020) | 32193296 | EUR, meta-analysis | 8.4% | (5.5%, 11.2%) | 31,707 | Higher PGS = greater thickness |
| cortical structure | Superior temporal gyrus thickness adjusted for global thickness | Grasby et al. (2020) | 32193296 | EUR, meta-analysis | 13.2% | (9.60%, 16.7%) | 30,739 | Higher PGS = greater thickness |
| cortical structure | Insula thickness adjusted for global thickness | Grasby et al. (2020) | 32193296 | EUR, meta-analysis | 8.6% | (5.6%, 11.7%) | 32,396 | Higher PGS = greater thickness |
| rate-of-change of brain structure | Average yearly rate-of-change in total brain volume | Bruower et al. (2022) | 35383335 | EUR, meta-analysis | 1.4% | (-5.4%, 7.9%) | 15,100 | Higher PGS = greater increase/less shrinkage |
| rate-of-change of brain structure | Average yearly rate-of-change in mean cortical thickness | Bruower et al. (2022) | 35383335 | EUR, meta-analysis | 4.2% | (-2.7%, 11.2%) | 15,100 | Higher PGS = greater increase/less shrinkage |
| rate-of-change of brain structure | Average yearly rate-of-change in total cortical grey matter volume | Bruower et al. (2022) | 35383335 | EUR, meta-analysis | 4.4% | (-2.5%, 11.3%) | 15,100 | Higher PGS = greater increase/less shrinkage |
| rate-of-change of brain structure | Average yearly rate-of-change in total cerebellar white matter volume | Bruower et al. (2022) | 35383335 | EUR, meta-analysis | 3.4% | (-6.7%, 6.5%) | 15,100 | Higher PGS = greater increase/less shrinkage |
| rate-of-change of brain structure | Average yearly rate-of-change in pallidum volume | Bruower et al. (2022) | 35383335 | EUR, meta-analysis | 4.8% | (-2.3%, 11.9%) | 15,100 | Higher PGS = greater increase/less shrinkage |
| rate-of-change of brain structure | Average yearly rate-of-change in putamen volume | Bruower et al. (2022) | 35383335 | EUR, meta-analysis | 7.6% | (-0.5%, 15.7%) | 15,100 | Higher PGS = greater increase/less shrinkage |
| rate-of-change of brain structure | Average yearly rate-of-change in nucleus accumbens volume | Bruower et al. (2022) | 35383335 | EUR, meta-analysis | 4.9% | (-2.1%, 12.0%) | 15,100 | Higher PGS = greater increase/less shrinkage |

*Note.* PGS = Polygenic score; N=GWAS sample size. SNP-based heritability estimates reported were estimated using LDSC and extracted from the original GWAS publications. For the cortical structure phenotypes, we reported the SNP-based heritability that corrected for average cortical thickness (See S7 in Grasby et al., 2022). For subcortical and cortical structure phenotypes, the N (sample size) was calculated as the largest sample size across SNPs in the GWAS summary statistics. For Neale Lab's (2019) and Mountford et al.’s (2021) GWASs, we estimated the LDSC SNP-based heritability for this table.
