# Supplementary material for: Polygenic Associations Between Motor Behavior, Neuromotor Traits, and Active Music Engagement in Four Cohorts
Source: Ann N Y Acad Sci. 2026 Feb 18;1556(1):e70191. doi: 10.1111/nyas.70191 (PMC12916079; doi:10.1111/nyas.70191)
Supplement: Supplementary file 3 — Supplementary Materials: nyas70191‐sup‐0003‐SuppMat.docx [file NYAS-1556-0-s001.docx]

Supplementary Information for: “Polygenic Associations between Motor Behaviour, Neuromotor Traits, and Active Music Engagement in Four Cohorts”

Henechowicz, T.L.^1,2,3,4,5^, Coleman, P.L.^3,6^, Gustavson, D.E.^5^, Mekki, Y.N.^3^, Nayak, S.^3,4^, Nitin, R.^3^, Scartozzi, A.C.^3,4^, Tio, E.S.^2^, van Klei, R.T.N.^2^., Felsky, D.*^2,7,8,9,10^, Thaut, M.H.*^1,11^, & Gordon, R.L.*^3,4,12,13^.

1. Music and Health Science Research Collaboratory, Faculty of Music, University of Toronto

2. Krembil Centre for Neuroinformatics, Centre for Addiction and Mental Health

3. Vanderbilt Genetics Institute, Vanderbilt University Medical Center

4. Music Cognition Laboratory, Department of Otolaryngology–Head and Neck Surgery, Vanderbilt University Medical Center

5. Institute for Behavioral Genetics, University of Colorado Boulder

6. Center for Digital Genomic Medicine, Vanderbilt University Medical Center

7. Department of Psychiatry, University of Toronto

8. Division of Biostatistics, Dalla Lana School of Public Health, University of Toronto

9. Rotman Research Institute, Baycrest Hospital, Toronto, ON

10. Department of Anthropology, University of Toronto

11. Temerty Faculty of Medicine, University of Toronto

12. Vanderbilt Brain Institute, Vanderbilt University

13. Department of Psychology, Vanderbilt University

*=co-supervision and co-senior authorship

**Table of Contents**

[1. Supplementary Methods 2](#_Toc212463616)

[1.1. Canadian Longitudinal Study on Aging 2](#_Toc212463617)

[1.2. Vanderbilt’s BioVU Repository 4](#_Toc212463618)

[1.3. Wisconsin Longitudinal Study 6](#_Toc212463619)

[1.4. Vanderbilt’s Online Musicality Study 8](#_Toc212463620)

[2. Supplementary Table Descriptions 10](#_Toc212463621)

[3. Supplementary figures 11](#_Toc212463622)

[4. References 21](#_Toc212463623)

# Supplementary Methods

## Canadian Longitudinal Study on Aging

1.1.1. Phenotyping

The Canadian Longitudinal Study on Aging is a cohort study of N=26,622 individuals with genotyped data available in release 3.0. Active music engagement was measured using the GEN_MUSC_COM variable from the Canadian Longitudinal Study on Aging’s General Health questionnaire from the Comprehensive Baseline Assessment version 7.0 (1,2). In the set of questions on mental exercise, participants were asked: “About how much time do you spend doing each of the following activities, taking into account both work and leisure time?” for “Playing a musical instrument or singing in a choir” with the response options of “Every day...1”, “Several times a week...2”, “Several times a month...3”, Several times a year...4”, “Once a year or less...5.” Participants who did not know, did not answer, or refused to answer were excluded from the analyses. We binarized the outcome to “musically engaged cases” for those who played “Several times a year” or more frequent answers and “controls” for those who answered, “Once a year or less.”

1.1.2. Genetic data

The CLSA data release version 3.0 provides imputed genotyped data in GRCh38/hg38 that was imputed using the TOPMed r2 reference panel (1–3). The CLSA cohort conducted pre-imputation genetic quality control on the data of 26,622 individuals who were genotyped using the UK Biobank Axiom array for 794,409 SNPs.

i. Pre-imputation quality control

The CLSA followed standardized quality filtering procedures similar to the UK Biobank (see CLSA version 3.0, section 1.4.3(1). Briefly, these steps included subsetting participants into ancestral groups by projecting principal component analyses onto 1000 genomes phase 3 populations, conducting within-ancestry marker-based quality control, and conducting sample-based quality control procedures (see CLSA version 3.0, section 4.0(1)). As a result, 26,622 CLSA participants and 716,347 SNPs that passed quality control (i.e., genotype frequency discordance between batches, departure from Hardy-Weinberg equilibrium (HWE), discordance across control replicates, discordance between males and females), SNP missingness <0.05, MAF>0.0001), were included for the phasing and imputation using the University of Michigan Imputation Service. Pre-phasing was conducted using EAGLE2(4) and Minimac2(5), and imputation was carried out with the TOPMed reference panel version r2(3) in two batches of 13,310 and 13,312 CLSA samples. During the imputation process, the TOPMed Imputation Server updated the positions of the genotyped data to GRCh38/hg38 using liftOver.

ii. Post-imputation quality control

We excluded multi-allelic SNPs and SNPs with *r^2^*<0.8. As per CLSA’s sample-level quality control protocol, samples were removed if they were determined to be outliers for heterozygosity or genotype missingness and had mismatches between self-reported and chromosomal sex (2). To achieve an unrelated set of individuals, we removed individuals in pairs with 3rd-degree relationships or closer using the KING software version 2.1.3 (see CLSA data release 3.0. section 4.2(1). Next, SNPs were mapped from chromosome:base pair coordinates to rsIDs using dbSNP155 mappings for genome assembly GRCh38 (downloaded from <https://hgdownload.soe.ucsc.edu/gbdb/hg38/snp/dbSnp155.bb> on March 13, 2024). Lastly, we removed SNPs with minor allele frequency (MAF) <0.01 using PLINK2.0 (6) and we extracted participants with European genetic ancestry (cluster four matching with populations of European ancestry from CLSA’s sample quality control metrics). The final imputed genotyped dataset in GRCh38/hg38 had 22,198 individuals with complete phenotype data and 8,321,411 SNPs included in the analyses.

iii. Genetic ancestry principal components analysis

We used the top ten principal components (PCs) provided in the CLSA sample quality control metrics file (CLSA data release 3.0, sections 4.4 and 4.5(1). The CLSA cohort computed PCs using *FlashPCA* version 2.0 (7). The input data for the PCA included 87,848 unambiguous biallelic SNPs, which met the following criteria: MAF > 0.05, HWE *p*-value>1×10⁻⁶, and linkage disequilibrium pruning (*r²* < 0.1) within 1,000 kb windows, with a step size of 5 markers.

## Vanderbilt’s BioVU Repository

- - 1. Phenotyping

The Vanderbilt BioVU sample includes 6,150 individuals (aged between 18 and 89 years old, M=53, SD=16.4) containing musically active cases and population-matched controls whose data were extracted from the Synthetic Derivative (SD) database at the Vanderbilt University Medical Center (VUMC), approved by Vanderbilt Institutional Review Board for nonhuman subjects research (IRB #160302). Musically active cases (N=1,259) were identified using an algorithmic search of 4 keywords and 449 regular expressions (selected examples include “musician”, “vocalist”, “songwriter”, “drummer”, “plays the piano”, “playing the guitar”, “played violin”, “player of the cello”, “plays saxophone”, “flutist”, “plays the flute”, “player of oboe”, “accordion player”; the complete list is given in supplementary table 2 of Niarchou et al.’s (2021) phenotyping algorithm(8). Population-matched controls (N=4,891), retrieved from the same study, were matched for ethnicity, race, and the median age at record length and did not have any of the music-related keywords/regular expressions (8,9).

- - 1. Genetic data

We accessed imputed and processed genotyped data in GRCh37/hg19, which were originally prepared as a polygenic score replication sample for Niarchou et al.’s (2022) genome-wide association study of musical beat synchronization. As described in section F of the Supplementary Information (9), the genotyped data were extracted from Vanderbilt University Medical Centre’s BioVU Biobank, where blood samples were assayed using Illumina bead arrays (MEGAEX) containing more than 2 million markers.

i. Pre-imputation quality control

Pre-imputation quality control was conducted in individuals of European ancestry including SNPs had individual call rates>0.98 and no sex discrepancies (|Fhet|<0.2). For imputation, the data was imputed to the Haplotype Reference Consortium panel (4) using the Michigan Imputation Server (4)(9).

ii. Post-imputation quality control

The data was filtered to include biallelic SNPs with MAF=>0.005, *r*^2^>=0.3, and call rates >0.98. SNPs were also removed for imputation batch effects and removed when MAFs within ancestry had >0.1 difference from corresponding 1000 genomes MAFs. Additional filters were applied within European ancestry, keeping SNPs with MAF>0.01 and HWE *p-*value>10^-10^. The BioVU sample used for the previous study included N=6,152 individuals. However, we added an identity-by-descent (IBD) relatedness filter of 0.2 for this analysis, which removed two related individuals. The final imputed dataset (GRCh37/hg19) consisted of 6,150 unrelated individuals and 6,360,678 SNPs passing QC.iii. Genetic ancestry principal components analysis

We used *FlashPCA* version 2.0(7) to generate ten genetic ancestry PCs. Prior to PCA, the data was preprocessed in PLINK 1.9(10), to retain SNPs with MAF > 0.05, HWE *p*-value > 0.001, and missingness < 0.02. We also excluded strand-ambiguous and multi-allelic SNPs and SNPs in regions of high LD (see list here: <https://genome.sph.umich.edu/wiki/Regions_of_high_linkage_disequilibrium_(LD)>), the MHC region (chr6:25–35 Mb), and inversion regions (chr8:7–13 Mb; chr17:40–45 Mb). Next, LD pruning was applied twice at r² < 0.2 within 200 kb windows.

##

## Wisconsin Longitudinal Study

- - 1. Phenotyping

The Wisconsin Longitudinal Study is a longitudinal study of men and women who graduated from Wisconsin high schools in 1957 and one of their randomly selected siblings, with data spanning over 60 years that allows researchers to link recently collected genetic data to social, health, and demographic characteristics (11). We extracted phenotype data from release 13.07 of the WLS data. Two music engagement phenotypes were selected from the 2003–2005 wave of data collection for graduates and spouses, which were collected via the Social and Civic Participation module of the mail-in survey. The phenotypes included:

- “How often did you play a musical instrument when you were about 35?” (q7spd_3), which had the responses of “often”, “sometimes”, or “never”. We binarized this phenotype to “often” and “sometimes” (as cases for music engagement) versus “never” (controls).
- “During the past year, how many hours per month did you play a musical instrument?” (q7spd_1). Due to a largely skewed distribution, with most of the sample reporting 0 hours of practice per month, we binarized the phenotype to those who practice >0 hours per month (cases) and those who practice 0 hours per month (controls).
  - 1. Genetic data

We accessed publicly available Haplotype Reference Consortium imputed data in GRCh37/hg19 from Lee et al.’s (2018) GWAS of educational attainment (12). In summary, Lee et al. (2018) extracted genotype data from the Illumina Human Omni Express Bead Chip, which was available for 9109 individuals and 713,014 SNPs.

i. Pre-imputation quality control

373 non-European individuals were identified and removed by plotting principal components of individuals’ genotype data together with 1000 Genomes populations. Second, individuals were excluded for genotype missingness >0.05, mismatch between genetic and surveyed sex, mismatch between surveyed relationship data and genetic relatedness, and extreme homozygosity/heterozygosity values (Fhet > |0.03|). SNPs were removed if they had a call rate <0.05, HWE *p*-value <10^-5^, and MAF<0.01. SNPs were also removed if: (i) A/T or G/C alleles and a MAF>0.4, (ii) alleles that do not match the Haplotype Reference Consortium data, (iii) MAF discrepancy with the Haplotype Reference Consortium data >0.2, (iv) not available in the Haplotype Reference Consortium data. 8,527 individuals with 604,710 autosomal SNPs were included for imputation using the Haplotype Reference Consortium v1.1 European reference panel using the Michigan Imputation Server.

ii. Post-imputation quality control

We kept SNPs with *r^2^>*0.7 and MAF>0.01. We restricted our analysis to unrelated individuals by removing those with third-degree or closer relatives, based on IBD estimates from Herd et al.’s (2016) genetic quality control report, which used 94,261 autosomal SNPs and the KING-robust method in *SNPRelate* (11,13). The final genotyped imputed dataset (in GRCh37/hg19) with 6,189 unrelated individuals and 7,554,744 SNPs. N=4,605 individuals had complete data for age, sex, and both music engagement phenotypes and were included in the statistical analyses.

iii. Genetic ancestry principal components analysis

We used the PCs provided by WLS from Lee et al.’s (2018) GWAS, which were calculated using PLINK v1.97 (10). Prior to PCA, Lee et al. (2018) processed the genotyped data with PLINK v1.97 to include SNPs with a call rate>0.99, MAF>0.01, and *r^2^>*0.6, and exclude SNPs in long-range LD regions on chromosomes 5 (44-51.5 Mb), 6 (25-33.5 Mb), 8 (8-12 Mb) and 11 (45-57 Mb). The remaining SNPs were LD-pruned (*r^2^*<0.1 on a 1000kb window).

##

## Vanderbilt’s Online Musicality Study

- - 1. Phenotyping

Vanderbilt’s Online Musicality study is a worldwide sample of adults (aged 18 to 89 years) recruited via Internet sources. It included demographic data collection, saliva samples for genotyping, and phenotyping focused on musicality and language traits (14). Music engagement was measured using an average score of four questions relating to self-report music engagement, which included “How engaged with music are you?” which was adapted from prior studies on music creative achievement (15,16). It had the following responses: “I am not engaged with music at all”; “I am self-taught and play music privately, but I have never played, sung or shown my music to others”; “I have taken lessons in music, but I have never played, sung or shown my music to others”; “I have played or sung, or my music has been played in public concerts in my home town, but I have not been paid for this”; “I have played or sung, or my music has been played in public concerts in my home town, and I have been paid for this”; “I am professionally active as a musician”; “I am professionally active as a musician and have been reviewed/featured in the national or international media and/or have received an award for my musical activities.” The remaining three items were extracted from the Goldsmiths Musical Sophistication Index (17): “I can sing or play music from memory” (coded as “Completely Disagree”; “Strongly Disagree”; “Disagree”; “Neither Agree nor Disagree”; “Agree”; “Strongly Agree”; “Completely Agree”), “I have never been complimented for my talents as a musical performer” (coded as “Completely Disagree”; “Strongly Disagree”; “Disagree”; “Neither Agree nor Disagree”; “Agree”; “Strongly Agree”; “Completely Agree”), and “At the peak of my interest, I practiced______hours per day on my primary instrument…” with coding of “0 (or never played an instrument)”; “0.5”; “1”; “1.5”; “2”; “3–4”; “5 or more”. For the present study, we examined three music engagement outcomes, the average of the score four music engagement items with higher scores indicating greater music engagement in addition to the self-contained untransformed items of music practice as measured by “At the peak of my Interest, I practiced…”, and music achievement as measured by “How engaged with music are you…”.

- - 1. Genetic data

We accessed imputed genotyped data in GRCh37/hg19 from Gustavson et al.’s (2023) Vanderbilt Online Musicality study. As described in Gustavson et al.’s (2023) study, 1,865 individuals were genotyped by VANTAGE using the Illumina Expanded Multi-Ethnic Genotyping Array (MEGAEX, 2,036,060 markers) of which 1,805 individuals met genetic quality control criteria.

i. Pre-imputation quality control

As reported in Gustavson et al.’s (2023) supplementary methods, SNPs were removed with MAF<0.01, variant missingness>0.10, and sample missingness>0.15. Individuals were removed for heterozygosity (|F|>0.2) and mismatching imputed and expected sex. We retained a maximally unrated set, removing individuals in pairs with 3rd-degree related or greater (kinship > 0.125, estimated using PC-relate) (21). We included individuals with European genetic ancestry using genetic-ancestry principal components calculated using PC-air (18) with reference to HapMap3 CEU and TSI populations. The data were filtered again within European ancestry to remove samples with missingness>0.05 and SNPs with HWE *p*-value <1x10^-8^. Prior to imputation, variant coordinates were lifted from GRCh37 to GRCh38 and all SNPs that were not present in GRCh38 were removed. Then, the sample was aligned to TOPMed reference data (3) to correct strand mismatches. SNPs were removed based on a differing allele frequency of >0.2 from the reference data, palindromic SNPs with an allele frequency of>0.4, and non-matching alleles or positions. For imputation, 1,805 individuals with 862,419 SNPs were phased with Eagle v2.4 (4) and then imputed to TOPMed version r2 (3) using Minimac4 (5) on the TOPMed imputation server (19).

ii. Post-imputation quality control

SNPs were filtered using BCFtools (20) and kept if they had MAF>0.01 and *r^2^*>0.7. The imputed dataset was lifted from GRCh38 to GRCh37 for the present analyses. The final imputed genotyped dataset was in GRCh37/hg19 and had 1,559 unrelated individuals of European ancestry with complete phenotypes and 9,335,401 SNPs.

iii. Genetic ancestry principal components analysis

For genetic ancestry principal components, we extracted the top 10 PCs that were calculated using PC-air (18).

# Supplementary Table Descriptions

**Supplementary Table 1.** Description of music engagement phenotypes in cohorts with individual genotyped data.

**Supplementary Table 2.**List of discovery GWAS included in PGS analyses

**Supplementary Table 3.** Outputs from the 168 main effects models in individual cohorts

**Supplementary Table 4.** Influential observations and sensitivity analyses for the 168 models in individual cohorts

**Supplementary Table 5.** Evaluation of sex-interactions for the 168 models in individual cohorts

**Supplementary Table 6.** Source data for “Figure 2” expressed as standard mean differences and including the meta-analyses
**Supplementary Table 7.** Results of meta-analyses including all cohorts and the leave-one-cohort-out analyses

# Supplementary figures


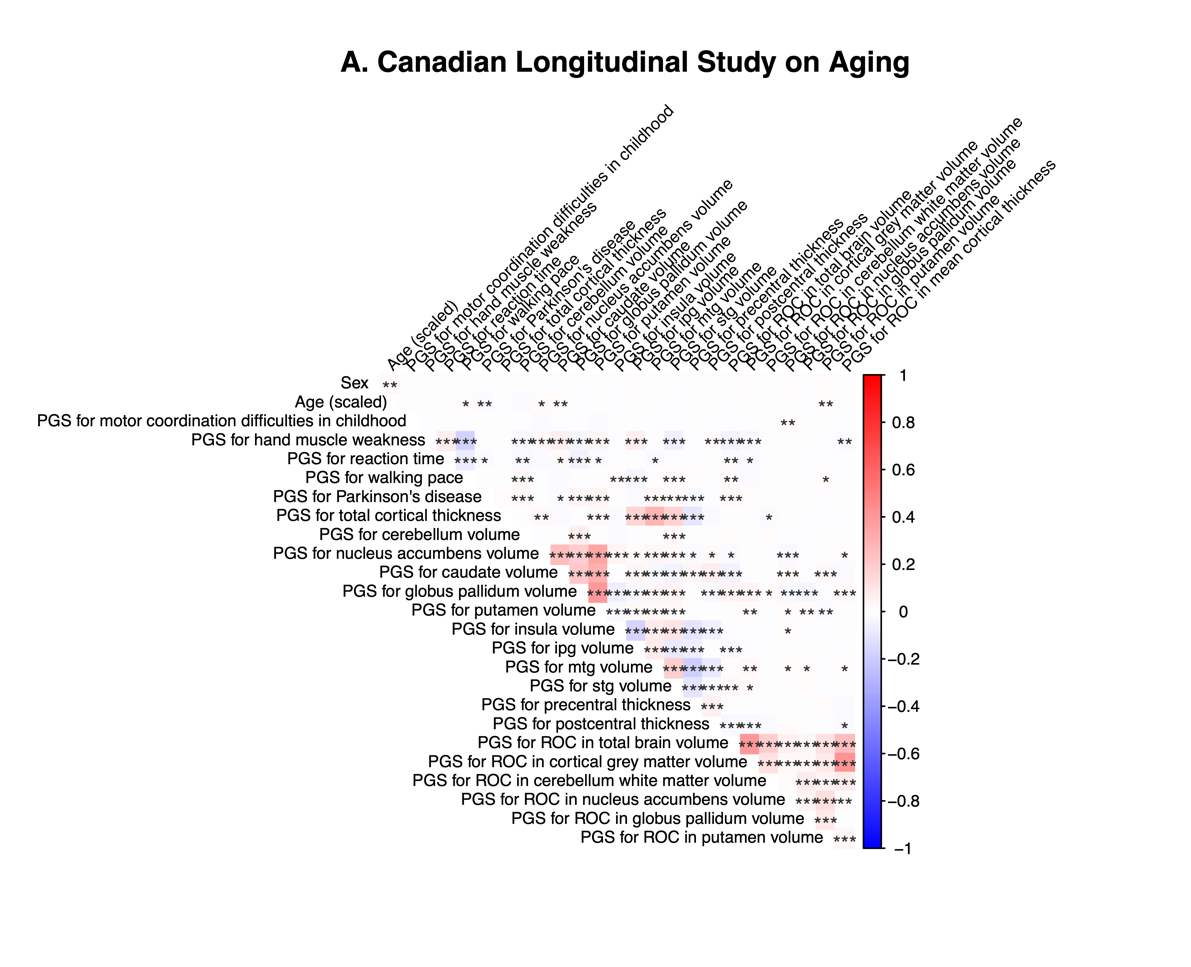

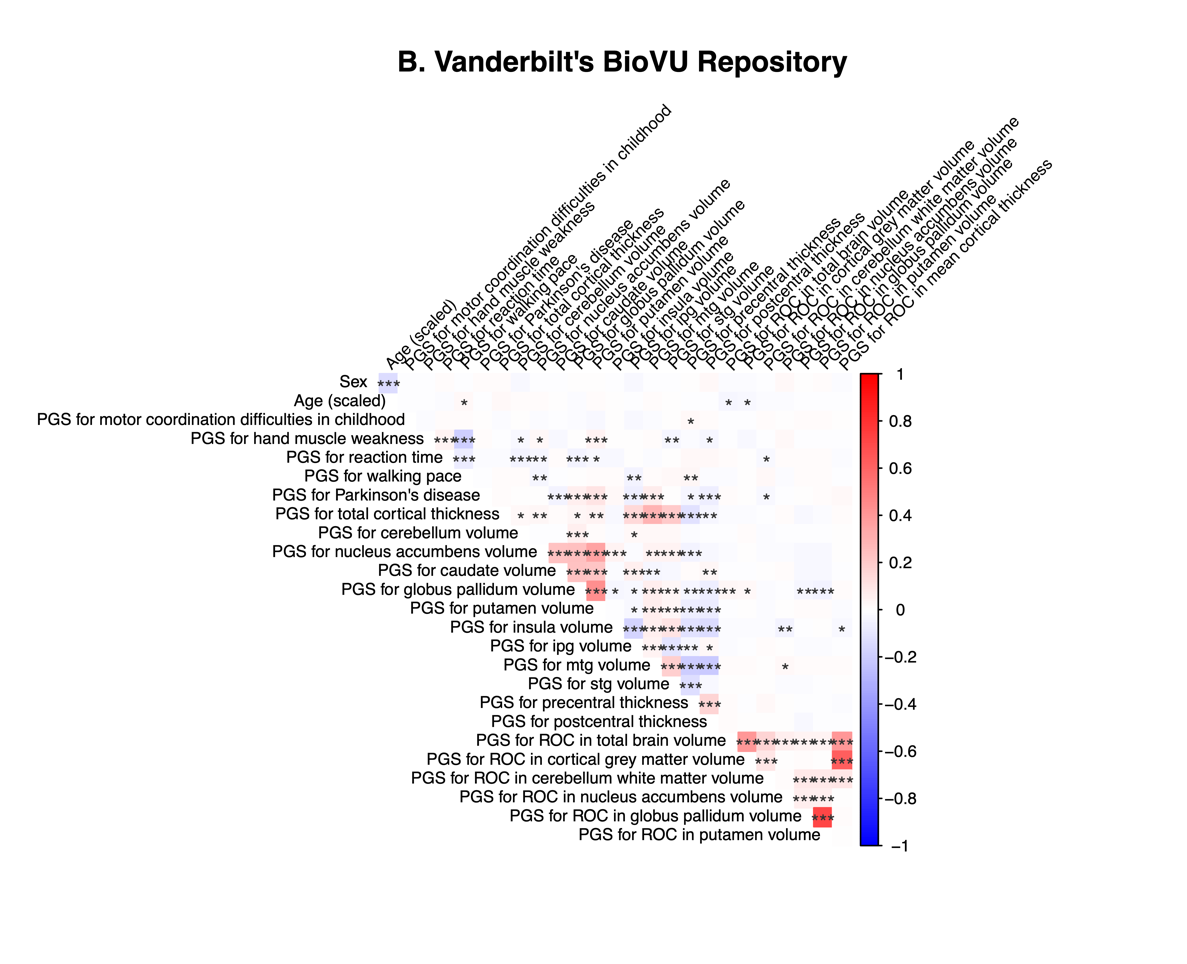


**
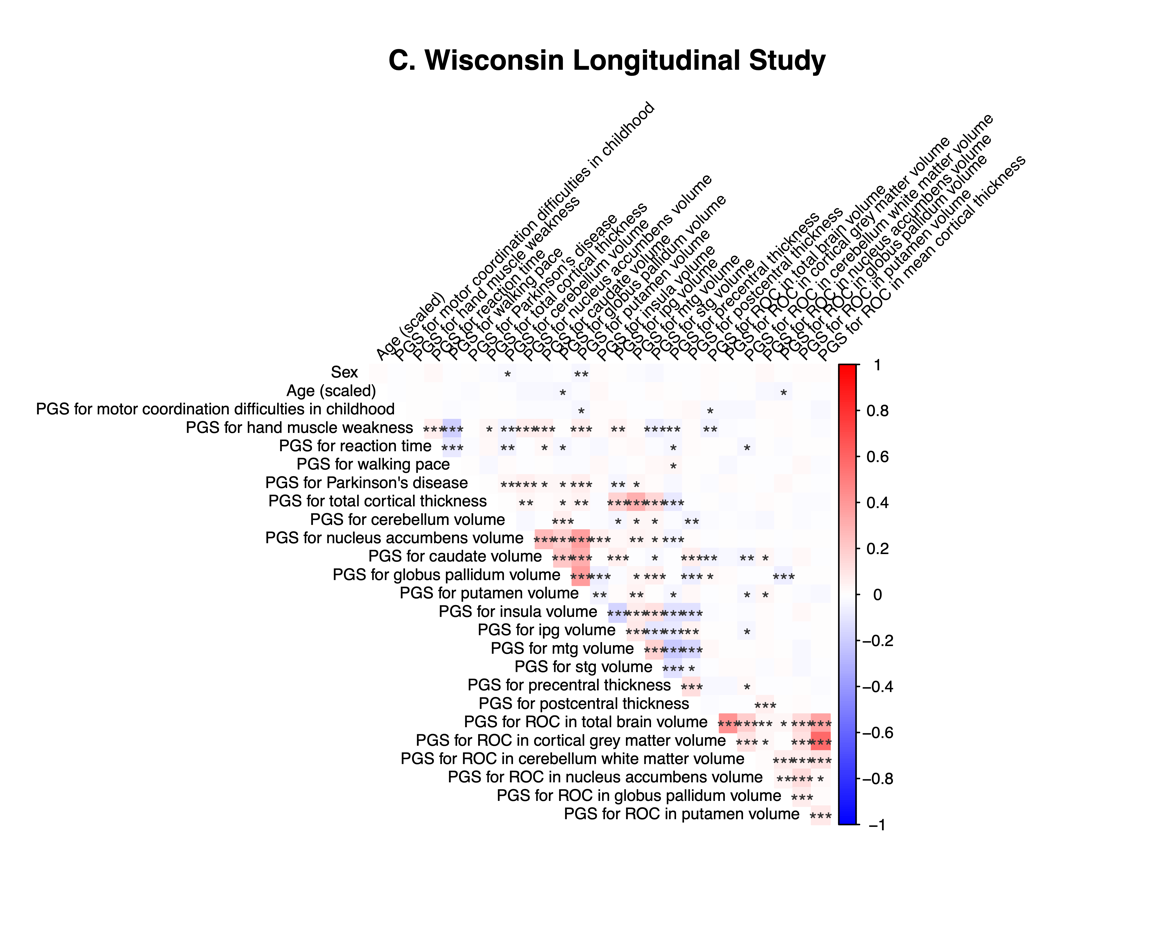


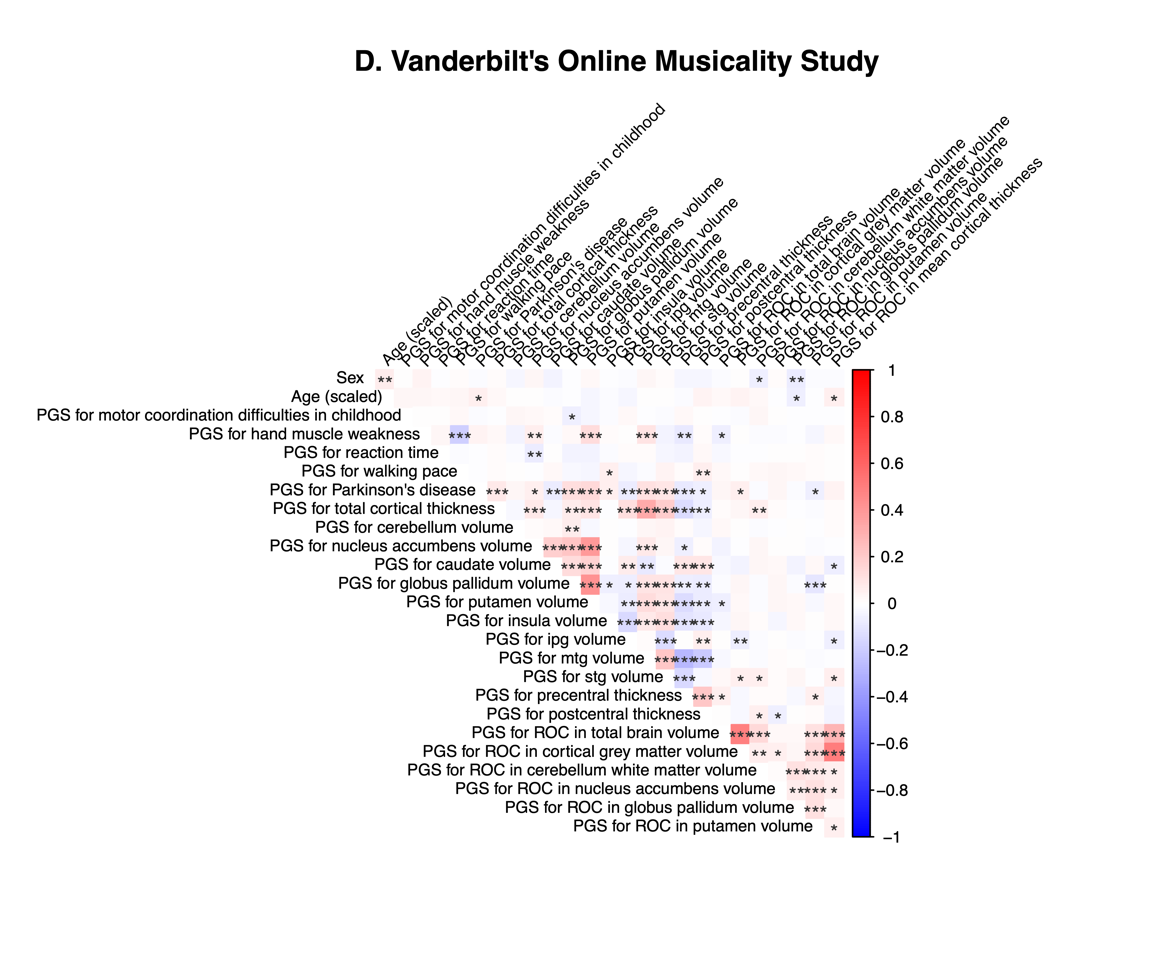
**

**Figure S1.** Correlation plots of predictor variables age, sex, and 24 polygenic scores for each cohort (A) Canadian Longitudinal Study on Aging, (B) Wisconsin Longitudinal Study, (C) Vanderbilt’s BioVU, and (D) Vanderbilt’s Online Musicality Study. Asterisks denote *p-*value significance of ****p<*0.001, ***p*<0.01, and **p*<0.05.

**
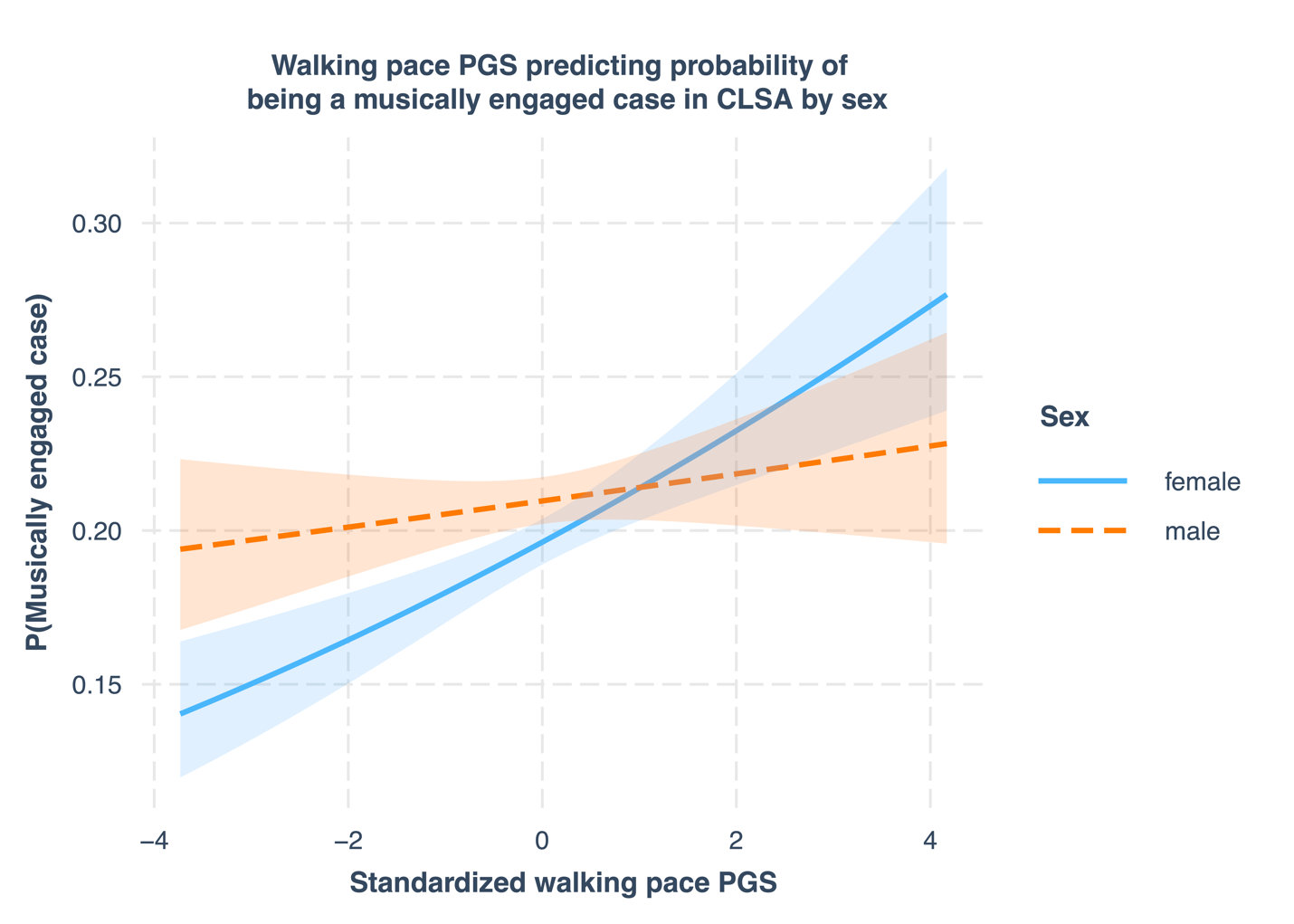
**

**Figure S2.** Interaction effect of sex by PGS for walking pace in the Canadian Longitudinal Study on Aging (CLSA). There was an interaction between PGS for walking pace and sex predicting music engagement (OR=0.92, 95% CI [0.86, 0.98], *p*=0.014, *q*FDR*=*0.34). Greater PGS for walking pace was associated with greater odds of music engagement in females (OR=1.11, 95%CI [1.06,1.17], *p*<0.001) although not significant in males (*p*>0.05).

**Figure S3.** Interaction effect of sex by PGS for walking pace on music engagement in Vanderbilt’s Online Musicality study. There was an interaction between PGS for walking pace and sex predicting music engagement (*b=*-0.21, 95% CI [-0.36, -0.05], *p*=0.008, *q*FDR*=*0.29). The PGS for walking pace was associated with increased music engagement in females (*b*=0.1, 95% CI [0.03, 0.18], *p*=0.007), although not significant in males (*p*>0.05).

**Figure S4.** Interaction effect of sex by PGS for walking pace on music practice in Vanderbilt’s Online Musicality study. When predicting music practice, there was an interaction between PGS for walking pace and sex (*b*=-0.26 95% CI [-0.45, -0.08], *p*=0.005, *q*FDR*=*0.29)*.* The PGS for walking pace was associated with increased music practice in females (*b*=0.13, 95% CI [0.04, 0.22], *p*=0.006) although not significant in males (*p*>0.05).

**Figure S5.** Interaction effect of sex by PGS for walking pace on music achievement in Vanderbilt’s Online Musicality study. When predicting music achievement, there was an interaction between PGS for walking pace and sex (*b=*-0.19, 95% CI [-0.36, (*b*=-0.19, 95% CI [-0.36, -0.03], p=0.018, qFDR=0.45)*.* The PGS for walking pace was associated with increased music achievement in females (*b*=0.11*,* 95% CI [0.03, 0.19]*, p*=0.007), although not significant in males (*p*>0.05).


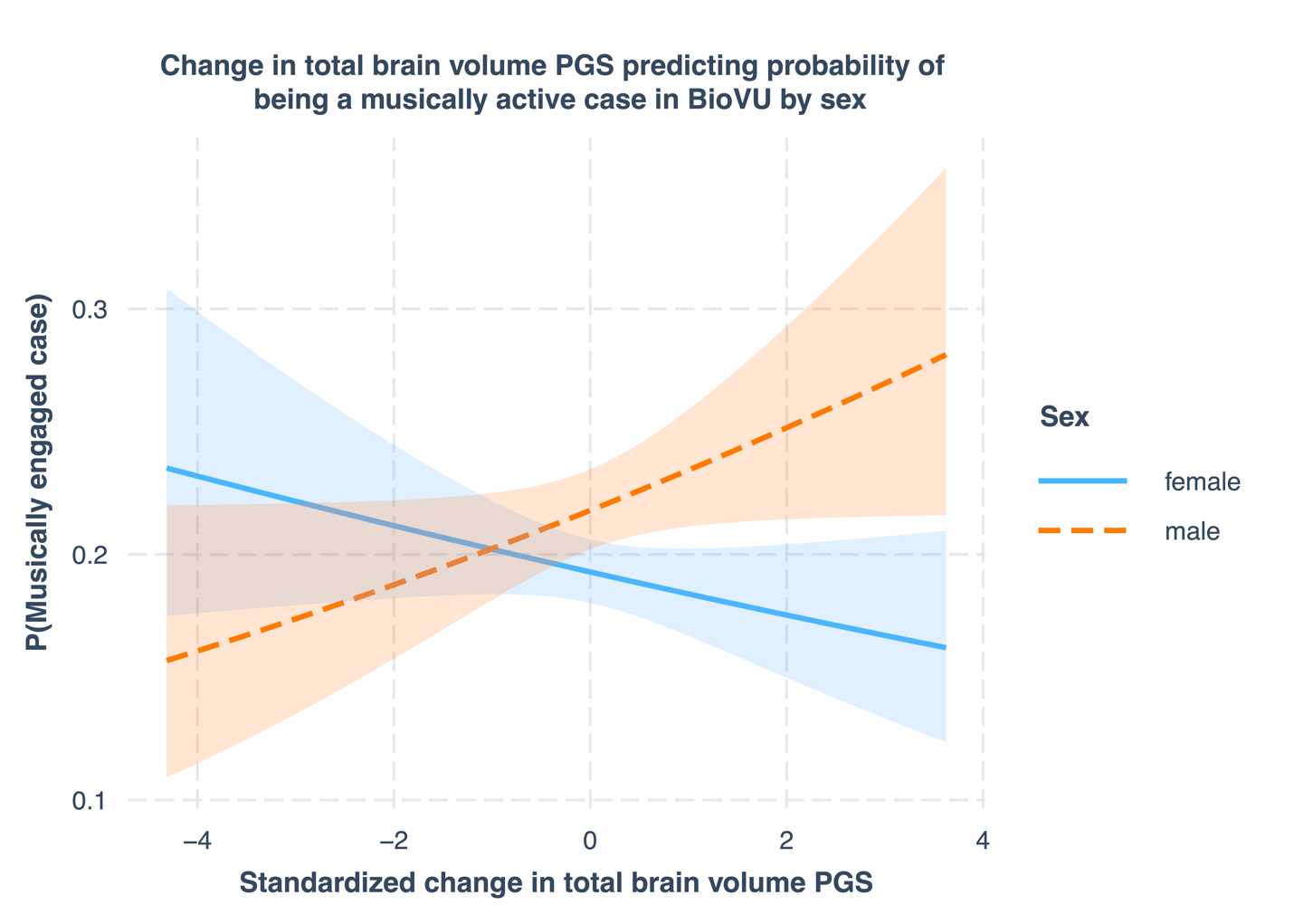


**Figure S6.** Interaction effect of sex by PGS for rate of change in total brain volume in Vanderbilt’s BioVU Repository. There was an interaction between the PGS for the rate of change in total brain volume and sex (OR=1.16, 95% CI [1.03, 1.32], *p*=0.02, *q*FDR=0.38). However, marginal effect models were not significant in males or females.


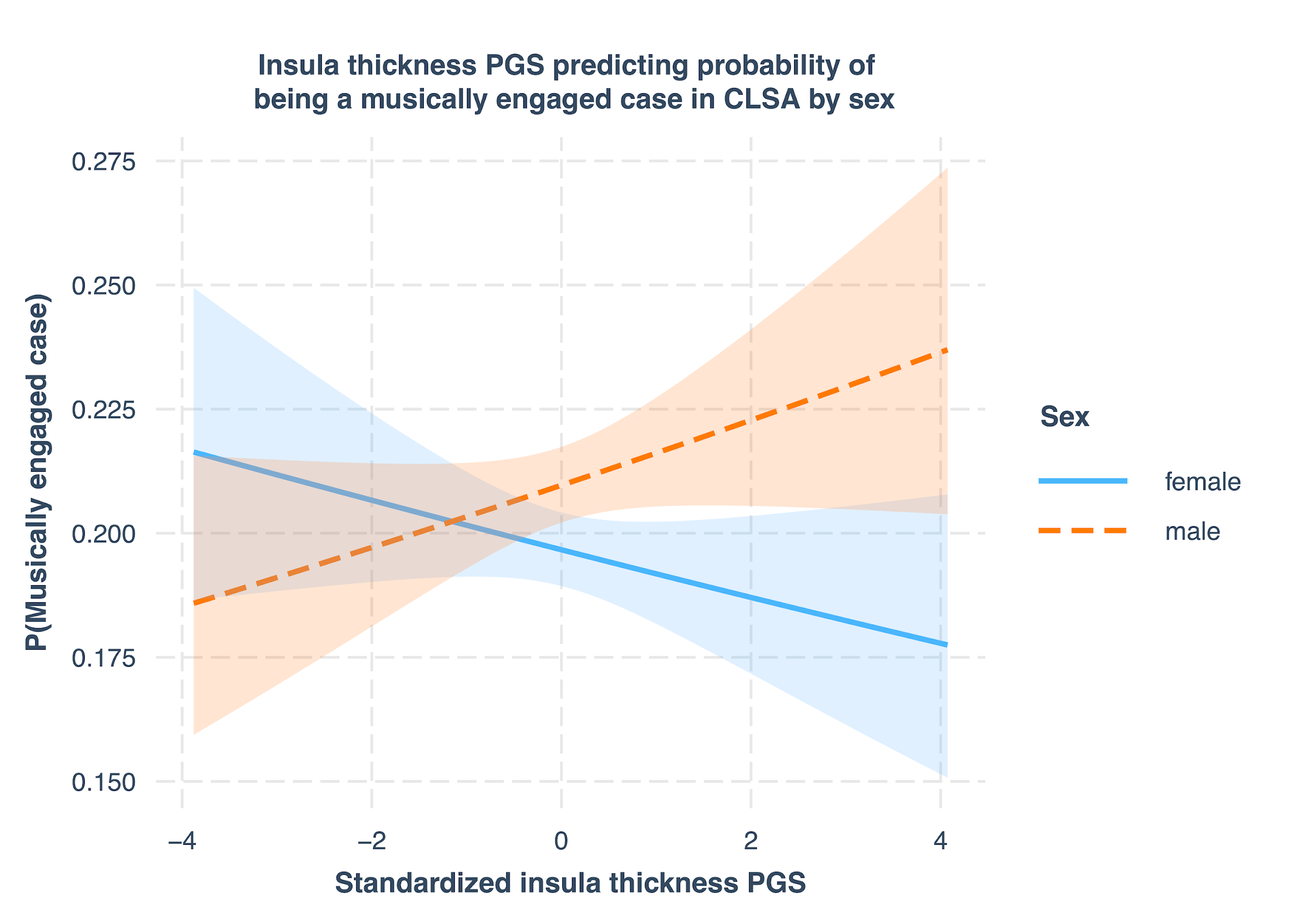


**Figure S7.** Interaction effect of sex by PGS for insula thickness in the Canadian Longitudinal Study on Aging. The model for PGS for insula thickness had an interaction with sex (OR =1.07, 95%CI [1.00,1.14], *p*=0.04, *q*FDR=0.44). However, marginal effect models were not significant in males or females.

**
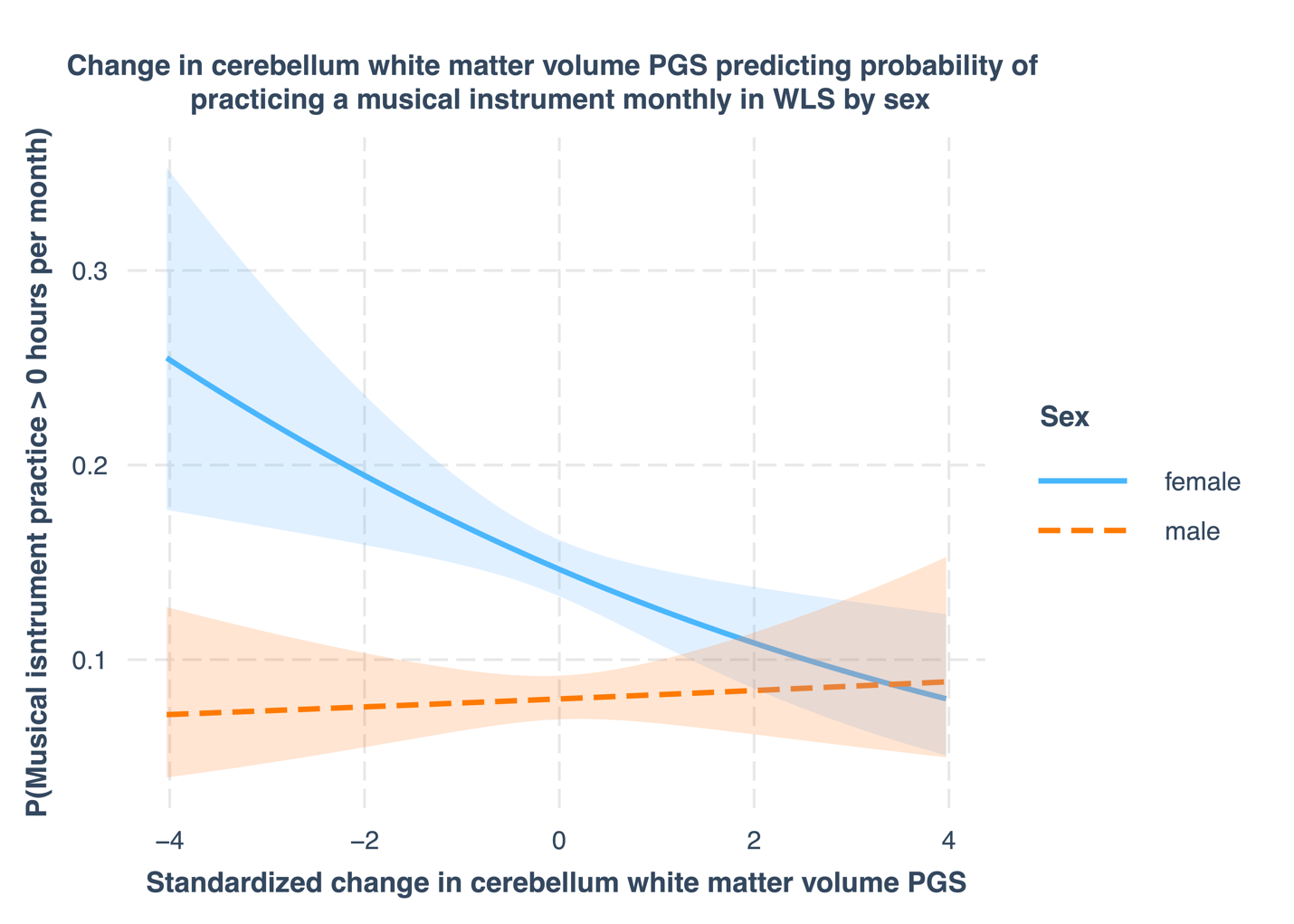
**

**Figure S8.** Interaction effect of sex by PGS for the rate of change of cerebellum white matter volume in the Wisconsin Longitudinal Study. The PGS for the rate of change in cerebellum white matter predicting music practice interacted with sex (OR =1.22, 95% CI [1.01, 1.48], *p*=0.04, *q*FDR=0.89). The PGS for the rate of change in cerebellum white matter was associated with decreased odds of practicing a musical instrument in females (OR = 0.84, 95% CI [0.75, 0.95], *p*=0.003) but not in males (*p*>0.05).

**Figure S9.** Interaction effect of sex by PGS for rate of change of mean cortical thickness in Vanderbilt’s Online Musicality study. There was an interaction between the PGS for the rate of change of mean cortical thickness and sex predicting music practice (*b*=0.20, 95% CI [0.02, 0.38], *p*=0.03, *q*FDR=0.50). However, marginal effect models were not significant in males or females.

# References

1. Forgetta V, Darmond-Zwaig C, Belisle A, Li R, Balion C, Wolfson C, et al. The Canadian Longitudinal Study on Aging.

2. Forgetta V, Li R, Darmond-Zwaig C, Belisle A, Balion C, Roshandel D, et al. Cohort profile: genomic data for 26 622 individuals from the Canadian Longitudinal Study on Aging (CLSA). BMJ Open. 2022 Mar 10;12(3):e059021.

3. Taliun D, Harris DN, Kessler MD, Carlson J, Szpiech ZA, Torres R, et al. Sequencing of 53,831 diverse genomes from the NHLBI TOPMed Program. Nature. 2021 Feb;590(7845):290–9.

4. Loh PR, Danecek P, Palamara PF, Fuchsberger C, Reshef YA, Finucane HK, et al. Reference-based phasing using the Haplotype Reference Consortium panel. Nat Genet. 2016 Oct 3;48(11):1443.

5. Fuchsberger C, Abecasis GR, Hinds DA. minimac2: faster genotype imputation. Bioinforma Oxf Engl. 2015 Mar 1;31(5):782–4.

6. Chang CC, Chow CC, Tellier LC, Vattikuti S, Purcell SM, Lee JJ. Second-generation PLINK: rising to the challenge of larger and richer datasets. GigaScience. 2015;4:7.

7. Abraham G, Inouye M. Fast Principal Component Analysis of Large-Scale Genome-Wide Data. PLOS ONE. 2014 Apr 9;9(4):e93766.

8. Niarchou M, Lin GT, Lense MD, Gordon RL, Davis LK. Medical phenome of musicians: an investigation of health records collected on 9803 musically active individuals. Ann N Y Acad Sci. 2021;1505(1):156–68.

9. Niarchou M, Gustavson DE, Sathirapongsasuti JF, Anglada-Tort M, Eising E, Bell E, et al. Genome-wide association study of musical beat synchronization demonstrates high polygenicity. Nat Hum Behav. 2022 Sept;6(9):1292–309.

10. Purcell S, Neale B, Todd-Brown K, Thomas L, Ferreira MAR, Bender D, et al. PLINK: a tool set for whole-genome association and population-based linkage analyses. Am J Hum Genet. 2007 Sept;81(3):559–75.

11. Herd P, Carr D, Roan C. Cohort Profile: Wisconsin longitudinal study (WLS). Int J Epidemiol. 2014 Feb;43(1):34–41.

12. Lee JJ, Wedow R, Okbay A, Kong E, Maghzian O, Zacher M, et al. Gene discovery and polygenic prediction from a genome-wide association study of educational attainment in 1.1 million individuals. Nat Genet. 2018 Aug;50(8):1112–21.

13. Visscher PM, Hemani G, Vinkhuyzen AAE, Chen GB, Lee SH, Wray NR, et al. Statistical Power to Detect Genetic (Co)Variance of Complex Traits Using SNP Data in Unrelated Samples. PLOS Genet. 2014 Apr 10;10(4):e1004269.

14. Gustavson DE, Coleman PL, Wang Y, Nitin R, Petty LE, Bush CT, et al. Exploring the genetics of rhythmic perception and musical engagement in the Vanderbilt Online Musicality Study. Ann N Y Acad Sci. 2023 Mar;1521(1):140–54.

15. Carson SH, Peterson JB, Higgins DM. Reliability, Validity, and Factor Structure of the Creative Achievement Questionnaire. Creat Res J. 2005;17(1):37–50.

16. Theorell TP, Lennartsson AK, Mosing MA, Ullén F. Musical activity and emotional competence – a twin study. Front Psychol [Internet]. 2014 July 16 [cited 2024 Nov 21];5. Available from: https://www.frontiersin.org/journals/psychology/articles/10.3389/fpsyg.2014.00774/full

17. Müllensiefen D, Gingras B, Musil J, Stewart L. The Musicality of Non-Musicians: An Index for Assessing Musical Sophistication in the General Population. PLOS ONE. 2014 Feb 26;9(2):e89642.

18. Conomos MP, Miller MB, Thornton TA. Robust inference of population structure for ancestry prediction and correction of stratification in the presence of relatedness. Genet Epidemiol. 2015 May;39(4):276–93.

19. Das S, Forer L, Schönherr S, Sidore C, Locke AE, Kwong A, et al. Next-generation genotype imputation service and methods. Nat Genet. 2016 Oct;48(10):1284–7.

20. Li H, Handsaker B, Wysoker A, Fennell T, Ruan J, Homer N, et al. The Sequence Alignment/Map format and SAMtools. Bioinforma Oxf Engl. 2009 Aug 15;25(16):2078–9.
